# HIV-1 Nef-induced cardiotoxicity through dysregulation of autophagy

**DOI:** 10.1038/s41598-017-08736-x

**Published:** 2017-08-17

**Authors:** Manish K. Gupta, Rafal Kaminski, Brian Mullen, Jennifer Gordon, Tricia H. Burdo, Joseph Y. Cheung, Arthur M. Feldman, Muniswamy Madesh, Kamel Khalili

**Affiliations:** 1 Department of Neuroscience, Center for Neurovirology and Comprehensive NeuroAIDS Center, Lewis Katz School of Medicine at Temple University, Philadelphia, PA USA; 2 Department of Medicine, Lewis Katz School of Medicine at Temple University, Philadelphia, PA USA; 3 Center for Translational Medicine, Lewis Katz School of Medicine at Temple University, Philadelphia, PA USA; 4 Cardiovascular Research Center, Lewis Katz School of Medicine at Temple University, Philadelphia, PA USA; 5 Department of Medical Genetics and Molecular Biochemistry, Lewis Katz School of Medicine at Temple University, Philadelphia, PA USA

## Abstract

Cardiovascular disease is a leading cause of co-morbidity in HIV-1 positive patients, even those in whom plasma virus levels are well-controlled. The pathogenic mechanism of HIV-1-associated cardiomyopathy is unknown, but has been presumed to be mediated indirectly, owing to the absence of productive HIV-1 replication in cardiomyocytes. We sought to investigate the effect of the HIV-1 auxiliary protein, Nef, which is suspected of extracellular release by infected CD4+ T cells on protein quality control and autophagy in cardiomyocytes. After detection of Nef in the serum of HIV-1 positive patients and the accumulation of this protein in human and primate heart tissue from HIV-1/SIV-infected cells we employed cell and molecular biology approaches to investigate the effect of Nef on cardiomyocyte-homeostasis by concentrating on protein quality control (PQC) pathway and autophagy. We found that HIV-1 Nef-mediated inhibition of autophagy flux leads to cytotoxicity and death of cardiomyocytes. Nef compromises autophagy at the maturation stage of autophagosomes by interacting with Beclin 1/Rab7 and dysregulating TFEB localization and cellular lysosome content. These effects were reversed by rapamycin treatment. Our results indicate that HIV-1 Nef-mediated inhibition of cellular PQC is one possible mechanism involved in the development of HIV-associated cardiomyopathy.

## Introduction

A large number of HIV-1 infected individuals develop cardiovascular diseases (CVD) with abnormal heart function causing significant morbidity and mortality^1, 2^. Antiretroviral therapy (ART) has dramatically improved the life span of HIV-1 infected patients, yet heart failure affects 7.4% of HIV-1 positive patients and is now a leading cause of death in HIV-1 infected persons^3^. HIV-1 patients show cardiomyopathy along with cardiac fibrosis^4–8^. HIV-1 can compromise cardiac function in the background of opportunistic infection, alcohol abuse, micronutrient deficiency and tobacco use^9, 10^, or native HIV-1 infected patients with or without ART. HIV-1 has been detected in the heart, yet the mechanisms of any such direct HIV-mediated cardiomyopathy, and progression towards heart failure, are unknown^11–13^.

Actively infected cells such as CD4^+^ T lymphocytes often release viral and cellular proteins with toxic activities that can be taken up by uninfected bystander cells leading to cell injury and death^14–16^. These observations have led to speculation that an indirect pathway potentially involving viral proteins may contribute to disease in some cell and tissue types including heart^17–21^. Nef is a 27 kDa HIV-1 auxiliary protein that localizes to the perinuclear space and distributes diffusely within the host cell membrane and cytosol^22–24^. This protein is relevant due to its important roles in HIV-1 replication, pathogenesis, reactivation and sexual transmission^25–27^. Even after ART, Nef is expressed by the proviral DNA and secreted to the extracellular environment^28^ and is present in high concentrations in the serum of HIV-1 positive patients, providing likely access to heart and other organs^14, 17, 29–32^. Nef may also be produced locally by HIV-1 infected immune cells that persist within the hearts of HIV-1 patients^33^. Additionally, Nef is sequestered within exosomes released from HIV-1 infected cells, and causes cell toxicity and apoptosis^14, 17, 31, 34^. The molecular and cellular events associated with cardiomyocyte injury and heart failure in the context of HIV-1 remains elusive.

Autophagy is a dynamic, self-digestive process important to cellular homeostasis that is responsible for regulating energy stores, clearing damaged organelles, and assuring protein quality control (PQC)^35, 36^. Various biological and physiological stimuli can dysregulate autophagy in myocardial cells causing adverse effects in heart muscle^37, 38^. HIV-1 manipulates autophagy in host cells to foster efficient viral production during an early stage of the viral life cycle, but at later stages may inhibit this pathway to promote host cell survival^39–43^. It is unknown whether such a mechanism contributes to cardiomyopathy in HIV-1 infected patients.

In the present study we tested the hypothesis that Nef directly impairs cardiomyocyte autophagy, viability and cell death. We found that Nef dysregulates autophagy in cardiomyocytes and abolishes autophagic flux. Also, we found that rapamycin, an mTOR pathway inhibitor, improves autophagy flux in Nef-expressing cardiomyocytes and promotes autophagosome-lysosome fusion by enhancing lysosomal biogenesis^44, 45^. Together, these findings suggest that rapamycin treatment can induce autophagy and improve function in Nef-bearing cardiomyocytes, raising the novel possibility of its use to ameliorate cardiomyopathy in HIV-1 infected patients.

## Results

### Detection of Nef in cardiomyocytes and circulating blood from HIV-1 positive patients and SIV infected animals

**Figure 1:**
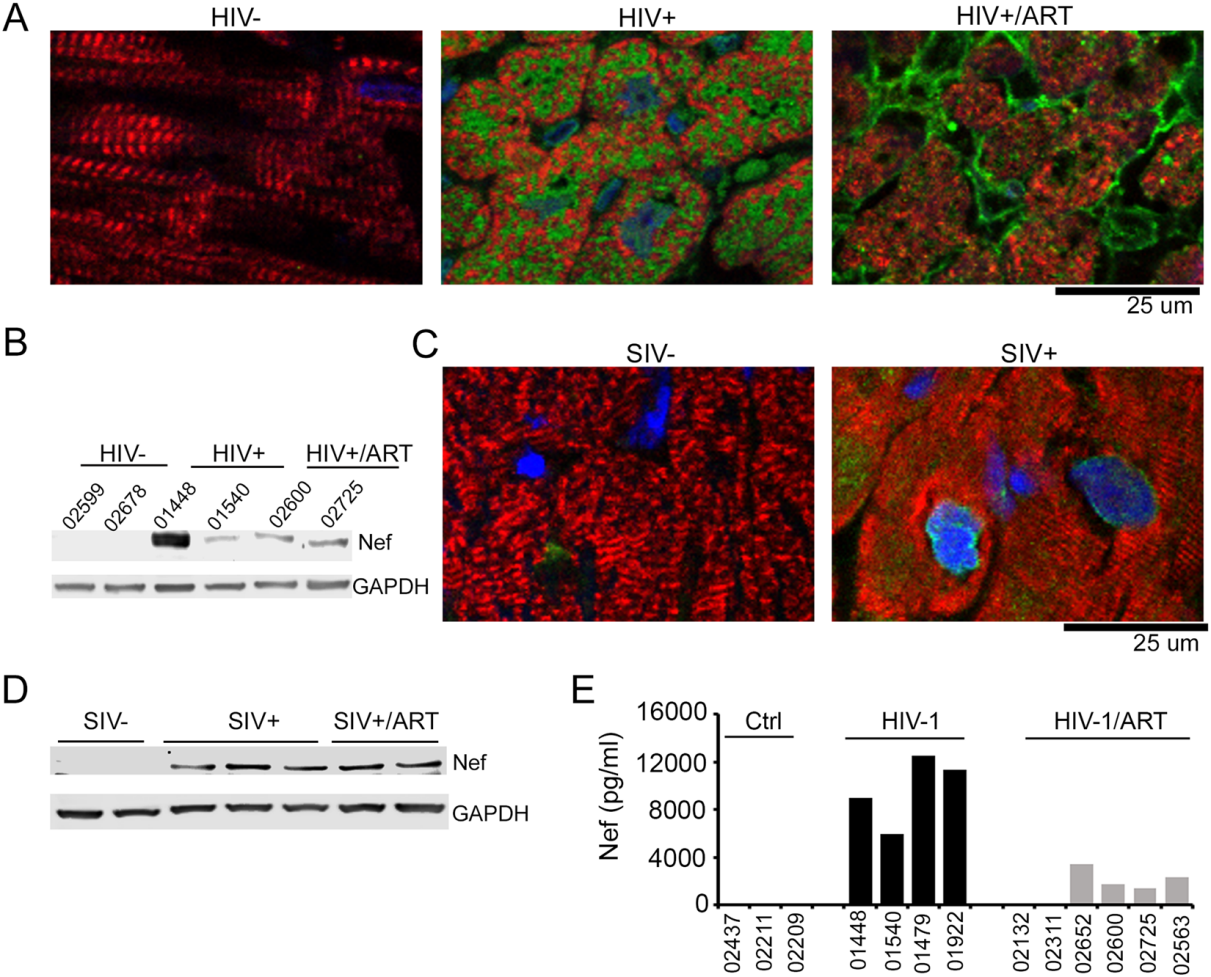
Nef protein is localized within cardiomyocytes in heart tissue of HIV-1 positive patients and SIV-infected macaques: (**A**) Representative images show the localization of Nef protein in heart tissue of HIV-1 positive patients treated (HIV+/ART) or not treated with ART (HIV+) but not in HIV-1 negative (HIV−) control. PFA-fixed human heart tissue sections were stained with antibodies directed against Nef (green) and cardiomyocyte marker protein Troponin I (TnI) (red). (**B**) Western blot analysis shows the presence of Nef protein in heart tissue lysates of HIV+ and HIV+/ART patients, but not HIV- subjects. The numbers represent the PROJID for each clinical samples obtained from NNTC. (**C**) Representative images show the presence of Nef protein in the cardiomyocytes of control (SIV−) or SIV-infected (SIV+) macaque heart tissue sections. Frozen heart tissue sections were fixed with acetone and stained with Nef-specific antibody (green) and TnI (red). (**D**) Western blot shows the presence of Nef protein in SIV-infected macaque hearts treated (SIV+/ART) and not treated (SIV+) but not in SIV− controls (SIV−). Blots were probed with antibody against SIV Nef (**E**) Nef was detectable in the serum of HIV-1+ patients with or without ART, but not control subjects, by ELISA.

As a first step, we investigated the presence of Nef in cardiac tissue from HIV-1 patients and SIV-infected macaques. Confocal microscopy images of formalin-fixed heart tissue sections from HIV-1 positive individuals and control HIV-1 negative subjects detected the presence of Nef within the HIV-1 positive individuals even in patients undergoing ART (Fig. 1A). This was further confirmed by Western blot analysis of protein extracts obtained from heart tissue of HIV-1 positive individuals (Fig. 1B). Similarly, hearts of SIV-infected and ART treated macaques showed the presence of SIV Nef protein in the cardiomyocytes (Fig. 1C and D). We also detected HIV-1 Nef protein by ELISA in the sera of HIV-1 positive patients, including those on ART, but not in HIV-1 negative subjects (Fig. 1E). These observations demonstrate that Nef accumulates in cardiomyocytes and is present in serum during HIV and SIV infection, even in the presence of ART.

### HIV-1 infection affects basal autophagy in cardiomyocytes

**Figure 2:**
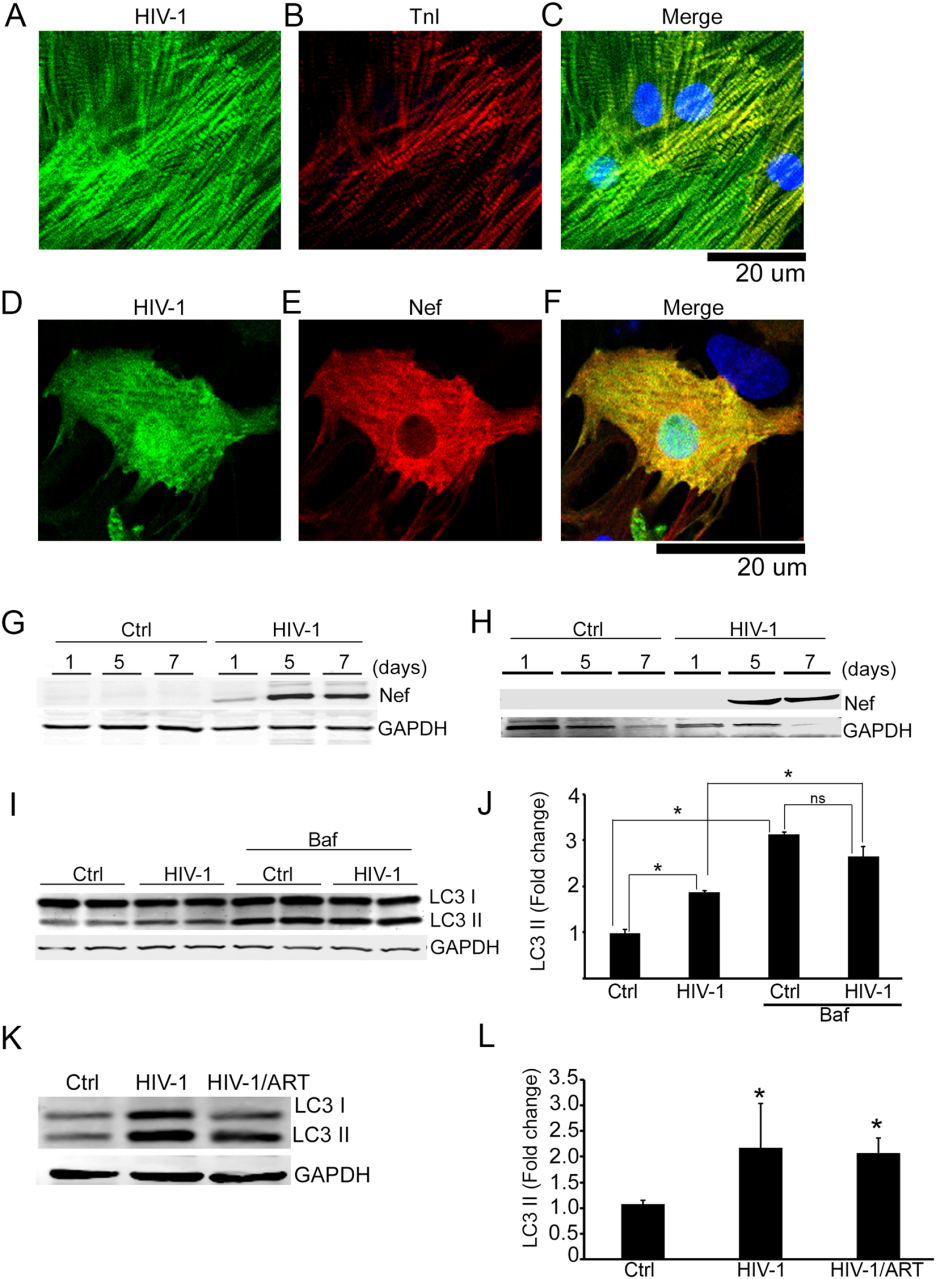
HIV-1 inhibits autophagy flux of cardiomyocytes. (**A**–**F**) Representative image shows infection in cardiomyocytes with pseudotyped HIV-1. NRVCs were infected with this virus (green) for 3 days and fixed with 4% PFA. Infected cells were stained with TnI, (**D**–**F**) pseudotyped HIV-1 infected cells (green) were stained with Nef antibody (red). (**G–H**) Western blots show progressive accumulation of Nef protein in total cell lysates (**G**) or culture filtrates (**H**) of pseudotyped HIV-1-infected cardiomyocytes at 1–7 days. **(I**,**J**) NRVC were treated with pseudotyped HIV-1 for 5 days and autophagy flux was measured with the autophagy inhibitor bafilomycin (Baf) (n = 6/group, *P < 0.05). Baf treatment elevated the LC3-II levels in both control and pseudotyped HIV-1 exposed cardiomyocytes, indicating that it inhibited autophagy flux. (**K**,**L**) Western blot and graph (n = 4–7) show increased expression of the autophagy marker protein LC3-II in human heart tissue lysates from HIV-1+ patients as well as in tissue lysates from HIV-1+ ART vs. those of HIV-1 negative (control) (*P < 0.05, significant vs Ctrl).

To better understand the interplay of HIV-1 with the process of autophagy in cells, neonatal rat ventricular cardiomyocytes (NRVCs) were infected with pseudotyped HIV-1-pNL4- 3GFP that allows expression of the viral proteins as well as the reporter GFP in the cells^46–48^. At 72 hours post infection, virtually all cells were GFP positive, indicating expression of the viral proteins in the infected cardiomyocytes (Supplementary Figure 1A). In addition, in infected NRVCs, GFP (Fig. 2A–C), and Nef (Fig. 2D–F) co-localized with the cardiomyocyte marker protein, troponin. Nef protein was detected in total cellular protein lysates as well as in culture media by Western blot (Fig. 2G and H). The detection of Nef in the culture medium was consistent with the earlier observations on the release of this protein by the infected cells^14, 49^.

Next, we assessed the effect of HIV-1 on autophagy in cardiomyocytes by the autophagy marker, LC3-II. During the initial stage of autophagy, free LC3-I is converted to LC3-II, its lipidated form, and remains membrane-associated with autophagy vesicles^36^. In the presence of HIV-1, cardiomyocytes contained significantly higher levels of LC3-II than the control cells (Fig. 2I and J), indicating that HIV-1 stimulates autophagy. Also, we investigated, in real time, the progression of autophagy using autophagy flux assay in which cells were treated with the autophagy inhibitor bafilomycin that blocks fusion of autophagosomes and lysosomes and causes accumulation of LC3-II in cells^50, 51^. Bafilomycin-treated HIV-1-infected cardiomyocytes showed no further increase in the LC3-II levels, suggesting that LC3-II accumulation may be the result of inhibition of autophagy at its terminal step (Fig. 2I and J). In addition, we found that inhibition of the terminal steps of autophagy by bafilomycin increases the level of Nef in the cytosol (Supplementary Figure 1B).

In hearts from HIV-1 positive patients, level of LC3 II were elevated compared to HIV-1 negative patients, irrespective of ART and found evidence for dysregulation of autophagy in cardiac tissue (Fig. 2K and L). Interestingly, treatment of cardiomyocytes with the conditioned medium from the HIV-1 infected peripheral blood mononuclear cells (PBMCs) is capable of dysregulating autophagy in the cells (Supplementary Figure 2A). These findings were further verified by additional studies using the Ad-tfLC3 reporter system^51, 52^, which allows for detection of real time autophagy process by highlighting autophagosome and autolysosome. Conditioned media from HIV-1-infected cells led to the accumulation of early autophagosome, depicted by accumulation of yellow autophagy puncta in the cells (Supplementary Figure 2B). The presence of Nef in the HIV-1 conditioned media was confirmed by Western blot (Supplementary Figure 2C).

### Nef causes cytotoxicity and inhibits autophagy flux in cardiomyocytes

Nef expression in NRVCs significantly increased cellular toxicity, decreased cellular viability and caused significant cell death in cardiomyocytes (Supplementary Figure 3). The effect of Nef on the autophagy process in cardiomyocytes was next examined. NRVCs were transduced with adenovirus expressing Nef (Supplementary Figure 4A and B). Nef formed aggregates which were localized within the perinuclear space of the cells (Supplementary Figure 4A). Interestingly, culture media of the Nef expressing cardiomyocytes contained Nef proteins, indicative of the extracellular release of this protein by cardiomyocytes (Supplementary Figure 4C). Conversely, treatment of the cardiomyocytes with a recombinant Nef showed intracellular presence of Nef (Supplementary Figure 4D).

**Figure 3:**
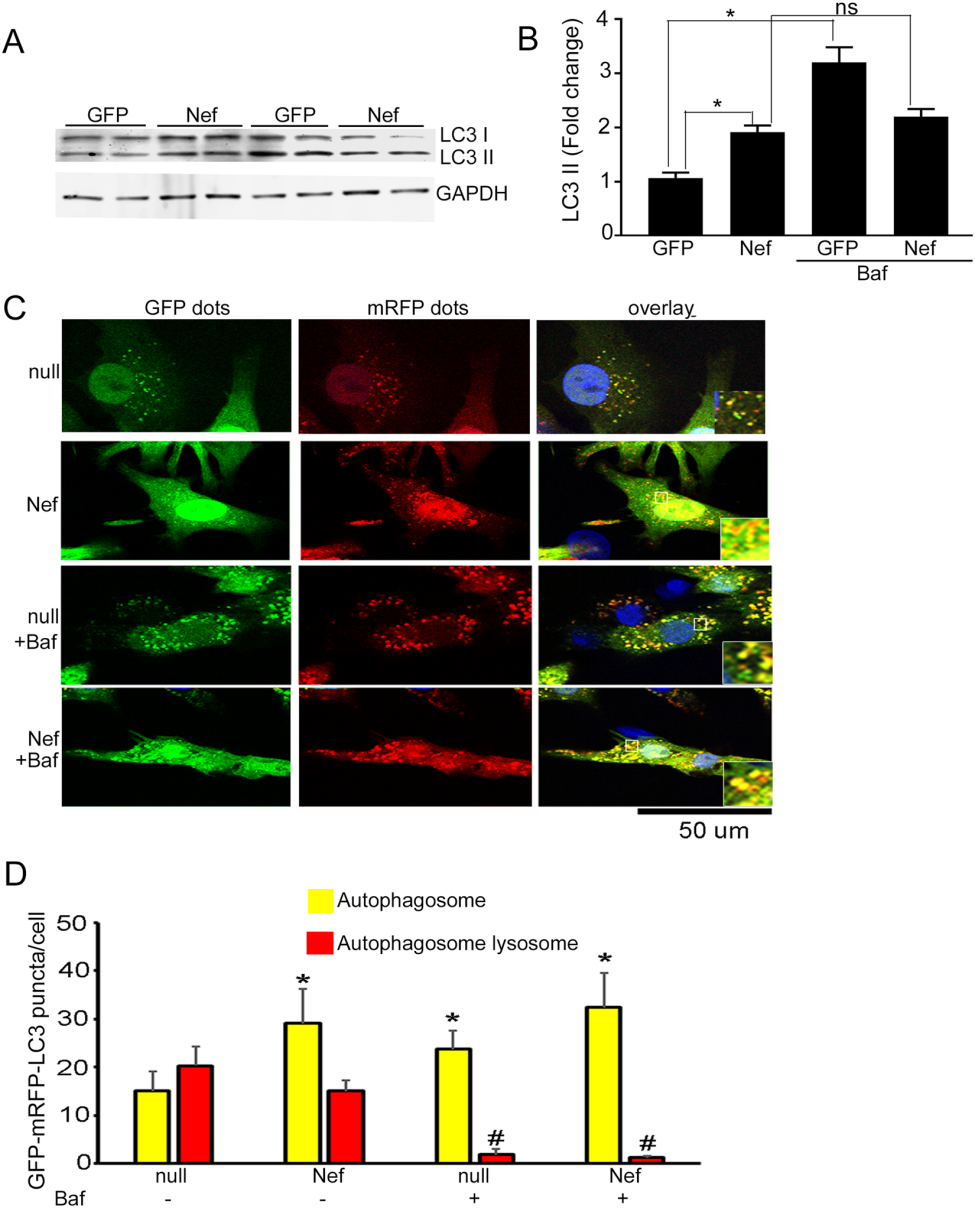
Nef blocks cellular autophagy at the terminal step. (**A**,**B**) Nef caused accumulation of autophagy marker protein LC3-II in cardiomyocytes. NRVCs were transduced with Ad-Nef or Ad-GFP for 48 hours and LC3 expression of was measured in total protein lysate by Western blot. Nef caused accumulation of autophagy marker protein LC3-II in cardiomyocytes. Autophagy flux assay was done using bafilomycin suggested that Nef causes blockage of autophagy flux in the cardiomyocytes (n = 6/group, *P < 0.05). (**C**,**D**) Progression of autophagy was monitored using autophagy reporter Ad-tfLC3. Representative images show the autophagosome (yellow) and autophagosome and lysosome fused puncta (red) in the cardiomyocytes transduced with Ad-tfLC3 (n = 4 wells/group, *P < 0.05, vs Ad-null, ^#^ *P* < 0.05, significant vs Ad-null).

NRVCs expressing Nef showed an increase in the level of LC3-II compared to cells expressing GFP (Fig. 3A and B). When the autophagy process was blocked by bafilomycin, there were no differences in LC3 II in NRVC expressing Nef suggesting that autophagy was inhibited in the Nef-expressing cardiomyocytes (Fig. 3A and B). This finding was further verified by an alternative method using an autophagy reporter system, Ad-tfLC3^52^. Consistent with the results from Western blot, we found that Nef-expressing cells have a higher accumulation of autophagosomes compared to control cells (Fig. 3C and D). These observations indicate that while Nef induces cellular autophagy, the terminal stages of autophagy that include degradation of the target protein(s) is aborted in the presence of Nef, it is the autophagic terminal (degradative) stage that is inhibited. Furthermore, we found that accumulation of large puncta corresponding to autophagosome in the Nef expressing cardiomyocytes after bafilomycin treatment suggesting that Nef inhibition of autophagosomes is halted at the maturation step.

**Figure 4:**
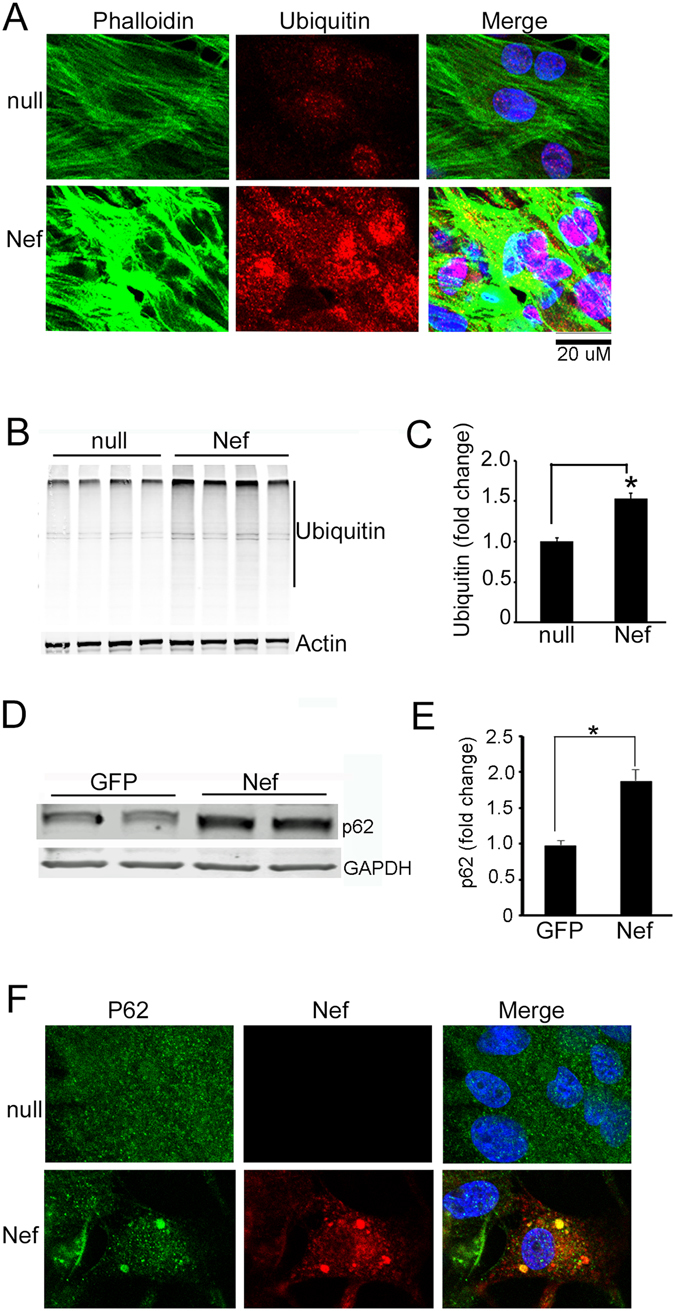
Nef increased accumulation of protein aggregates in cardiomyocytes. (**A**) Representative confocal images show accumulation of ubiquitin in the cardiomyocytes expressing Nef. NRVCs were transduced with control Ad-null or Ad-Nef for 48 hours, fixed with 4% PFA, and stained with ubiquitin antibody. (**B**,**C**) Western blot analysis confirms that ubiquitin levels are increased in the Nef-treated cardiomyocytes (n = 4g/group, *P < 0.05, significant difference between Ad-null and Ad-Nef). (**D**,**E**) NRVCs were transduced with Ad-Nef or Ad-GFP for 48 hours and total protein lysates were analyzed by Western blot using SQSTM1/p62-specific antibody; p62 levels are significantly increased in Nef-expressing cardiomyocytes (n = 6/group, *P < 0.05, significant difference between Ad-GFP and Ad-Nef). (**F**) Representative confocal images indicate that Nef and p62 are colocalized in perinuclear aggregates in Nef-expressing cells. NRVCs were transduced with Ad-null or Ad-Nef for 48 hours and cells fixed with 4% PFA and stained with Nef- and p62-specific antibodies.

In follow-up experiments, we examined whether Nef-mediated inhibition of autophagy results in the accumulation of protein aggregates. The level of protein aggregates in Nef-expressing cardiomyocytes was determined by immunocytochemistry using an anti-ubiquitin antibody and a dramatic increase in the level of ubiquitin positive aggregates in comparison to the control cells was found (Fig. 4A). In agreement, results from Western blot showed that Nef caused accumulation of poly-ubiquitin protein in the cardiomyocytes (Fig. 4B,C). Earlier studies suggested that p62 plays a significant role in the recognition of autophagic cargo and assists their autophagy-mediated degradation process^53^. During blockage of autophagy, p62 forms aggregates with the LC3 and accumulates in the cytosol^43^. Therefore, we determined the level expression of p62 protein in Nef-treated cardiomyocytes and found that the level of p62 was significantly upregulated in Nef-expressing cardiomyocytes (Fig. 4D and E). Confocal images demonstrated the perinuclear localization of aggregates harboring p62 and Nef (Fig. 4F).

### Interaction of Nef with autophagy regulators

**Figure 5:**
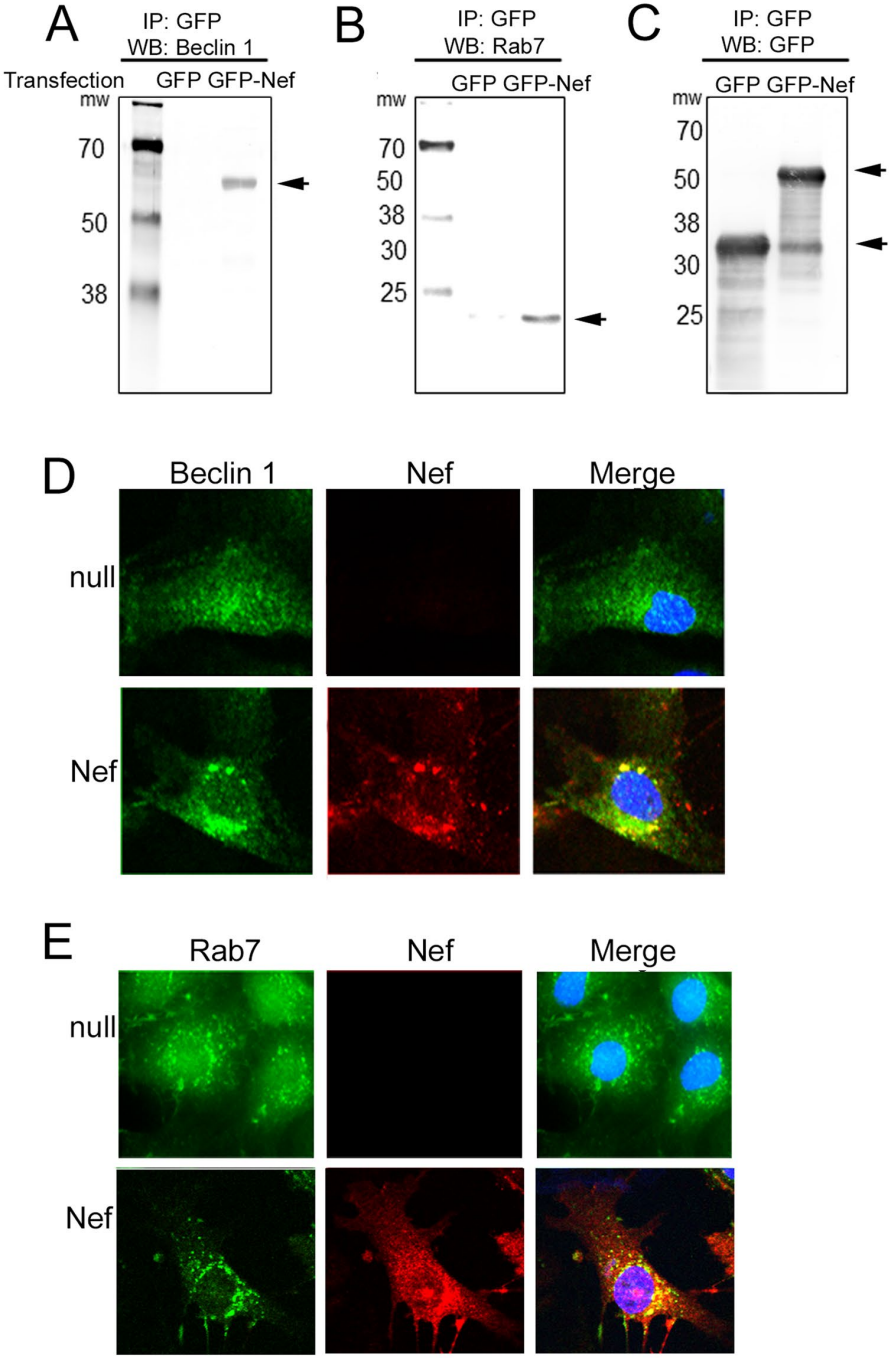
Nef binds to autophagy maturation factors Beclin 1 and Rab7 and colocalizes to the perinuclear space. (**A**–**C**) Nef interacts with the autophagy maturation factors Beclin 1 and Rab7, shown by immunoprecipitation. Nef-GFP fusion protein or GFP alone were expressed in the HEK293T cells for 3 days, lysates were immunoprecipitated using GFP-specific antibody, and immunoprecipitates were analyzed by Western blot with Beclin 1- (Panel A), Rab7- (Panel B), and GFP-specific (Panel C) antibodies. (**D**,**E**) Confocal images show that Nef colocalizes with Rab7 and Beclin 1 in perinuclear punctae. Cardiomyocytes were transduced with Ad-null or Ad-Nef for 48 hours, and cells were fixed and stained Beclin 1-, Rab7- and Nef-specific antibodies.

To further investigate Nef-mediated inhibition of autophagy, we examined potential interaction of Nef with autophagosome maturation factors, including Beclin-1. Co-immunoprecipitation revealed the interaction of Beclin-1 with GFP-Nef, but not GFP (Fig. 5A). Confocal microscopy demonstrated co-localization of Nef and Beclin 1 in cardiomyocytes and formation of aggregates at the perinuclear space (Fig. 5D). In addition, we found that Nef interacts with the autophagosome maturation factor Rab7, a protein which also plays a critical role in the fusion of autophagosomes and lysosomes and the completion of autophagy (Fig. 5B). Microscopic imaging showed subcellular localization of Rab7 is significantly altered in the presence of Nef. In the absence of Nef, Rab7 forms isolated punctate structure throughout the cytosol; whereas in the presence of Nef, Rab7 is absent in the isolated punctate yet detected in the aggregates with Nef within the perinuclear space (Fig. 5E). Furthermore, the non-aggregate forms of Rab7 were significantly decreased in Nef expressing cardiomyocytes, while the aggregates that contain Rab7 showed an increase in Nef (Supplementary Figure 5). In agreement with these observations, infection of cardiomyocytes with HIV-1 pseudotyped altered subcellular localization of Rab7 in the infected cells (Supplementary Figure 6). These observations suggest that Nef, by dysregulating autophagy regulators including Beclin-1 and Rab7, interferes with the process responsible for the maturation of autophagosome. It is noteworthy to mention that expression of Nef was accompanied by an increase in the level of phosphorylation of AKT (Supplementary Figure 7), an event which may also contribute to the autophagosome maturation and autophagy in cardiomyocytes^54^.

### Rapamycin restores autophagy in Nef-expressing cardiomyocytes

Earlier studies suggested that autophagy can be modulated by pharmacological inducers such as rapamycin, which, in turn, may protect cardiomyocytes from injury during stress conditions^55–57^. Here, we tested whether rapamycin treatment can neutralize the toxic effect of Nef in cardiomyocytes and restore autophagic flux in these cells.

**Figure 6:**
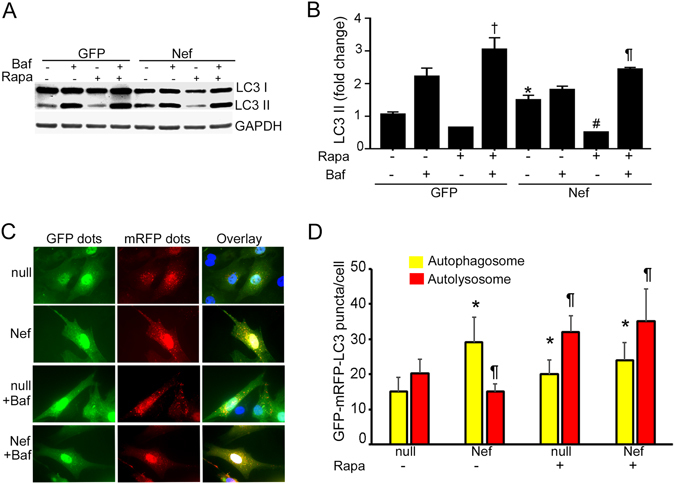
Rapamycin treatment restores the autophagy flux of Nef-expressing cardiomyocytes. (**A,B**) Western blot analysis with LC3-specific antibody shows Nef-expressing cells have improved autophagy flux after rapamycin treatment compared to vehicle treated cells. NRVCs were transduced with Ad-Nef or Ad-GFP for 48 hours, treated with rapamycin for 12 hours, with bafilomycin added 4 hours before harvesting cell lysates (n = 6/group, *P < 0.05, significant difference between Ad-GFP and Ad-Nef, ^#^ *P* < 0.05, significant difference between Ad-Nef and Ad-Nef treated with rapamycin, ^¶^ *P* < 0.05, significant difference between Ad-Nef treated with bafilomycin and Ad-Nef treated with bafilomycin + rapamycin). (**C**,**D**) Autophagic flux of rapamycin-treated cells was analyzed with autophagy reporter system Ad-tfLC3. Microscopy imaging shows autophagosomes (yellow) and autolysosomes (red) puncta in the cardiomyocytes. The graph shows the ratio of red and yellow puncta in rapamycin-treated cardiomyocytes (n = 4 wells/group, *P < 0.05, significant difference between Ad-null, Ad-Nef, Ad-null treated with rapamycin and Ad-Nef treated with rapamycin, (n = 4 wells/group, ^¶^ *P* < 0.05, significant difference between Ad-null with Ad-Nef, Ad-null treated with rapamycin and Ad-Nef treated with rapamycin).

**Figure 7:**
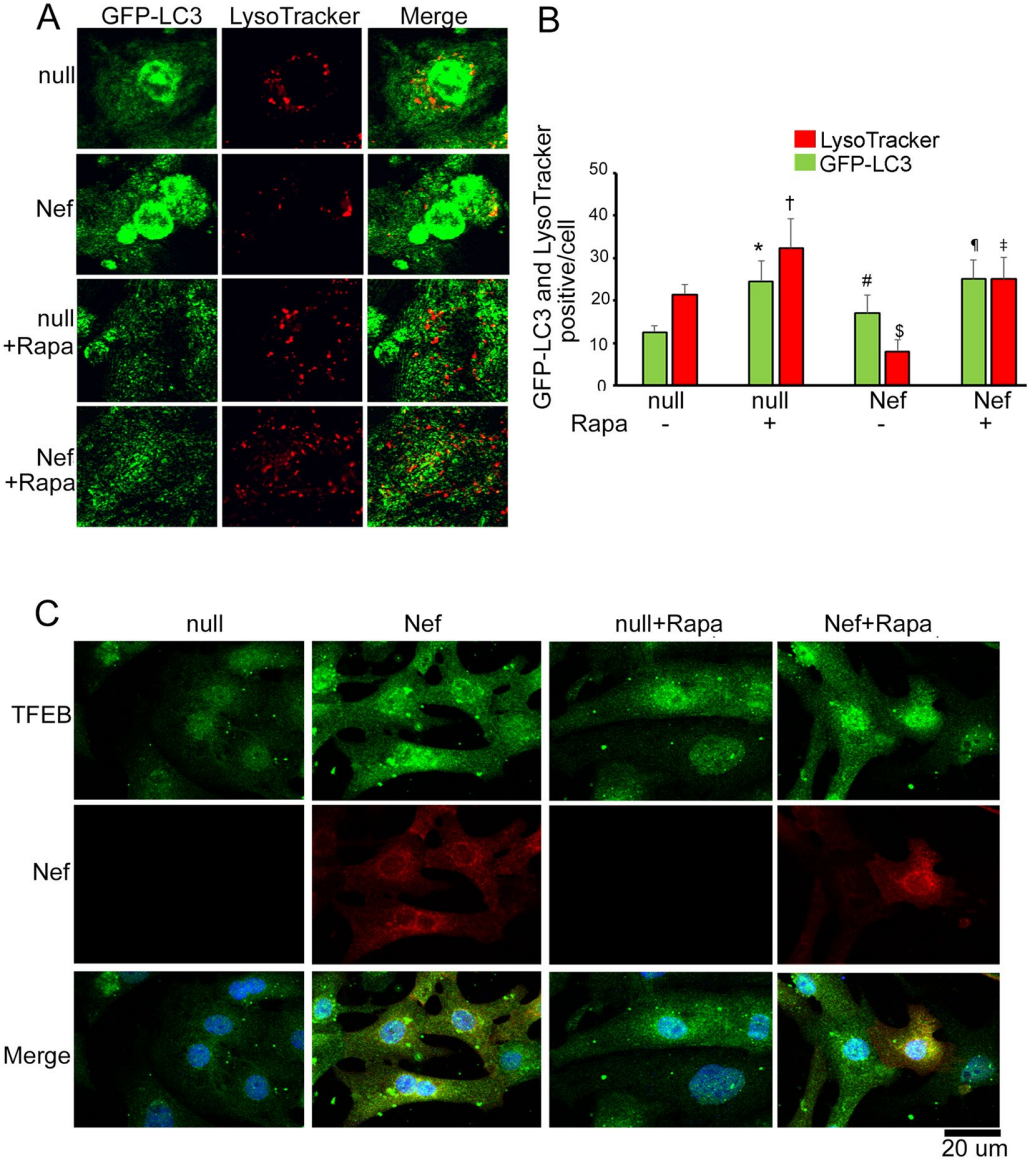
Rapamycin treatment restored the lysosome content of cardiomyocytes. (**A**) NRVCs were transduced with Ad-GFP-LC3 for 48 hours, incubated with LysoTracker (red), and observed by confocal microscopy. Representative image shows the localization of lysosome (red) and autophagy puncta (green) in Nef expressing cells. (**B**) Quantification of lysosomes and autophagosomes in cardiomyocytes (n = 4 wells/group, *P < 0.05, vs Ad-null; ^†^ *P* < 0.05, vs Ad-null; ^#^ *P* < 0.05, vs Ad-null; ^$^ *P* < 0.05, vs Ad-null; ^¶^ *P* < 0.05, vs Ad-null and Ad-Nef; ^‡^ *P* < 0.05, vs Ad-Nef). (**C**) Representative images showed that rapamycin treatment reduced cytosolic accumulation of TFEB protein in Nef transduced cardiomyocytes. NRVCs were transduced with Ad-null or Ad-Nef for 48 hours and treated with rapamycin for 12 hours. Cells were fixed with 4% PFA and immunocytochemistry was done with TFEB (green) and Nef (red) antibodies respectively.

Rapamycin treatment improved the cellular morphology by reducing apoptotic body formation (Supplementary Figure 8A) and cell death (Supplementary Figure 8A and B), and decreased Nef-induced cytotoxicity **(**Supplementary Figure 8C). Viability was also significantly improved in Nef-expressing cardiomyocytes in presence of rapamycin (Supplementary Figure 8D and E). These data suggest that rapamycin-mediated induction of autophagy has a beneficial effect on Nef-expressing cardiomyocytes. In addition rapamycin treatment prevented the accumulation of LC3-II in Nef-expressing cells and significantly improves autophagic flux (Fig. 6A and B). Rapamycin-mediated induction of autophagic flux was verified by the autophagy reporter system assay Ad-tfLC3. Rapamycin treatment improved the autophagy flux of control cells as well as Nef-expressing cells (Fig. 6C and D). To evaluate if Nef affects the lysosome content of cells, we used LysoTracker, an indicator dye for imaging of lysosome content in live cells. Nef-expressing cells showed a reduction of LysoTracker puncta (red) and increase of autophagy puncta (green) compared to control cells (Fig. 7A and B). Rapamycin treatment improved the lysosome content in the Nef-expressing cardiomyocytes (Fig. 7A and B). As subcellular positioning of TFEB is critical in the cellular lysosome content, we found that Nef increased accumulation of TFEB containing aggregate in the cytosol (Fig. 7C). Rapamycin treatment removed the TFEB+ cytosolic aggregates from Nef transduced cardiomyocytes (Fig. 7C).

**Figure 8:**
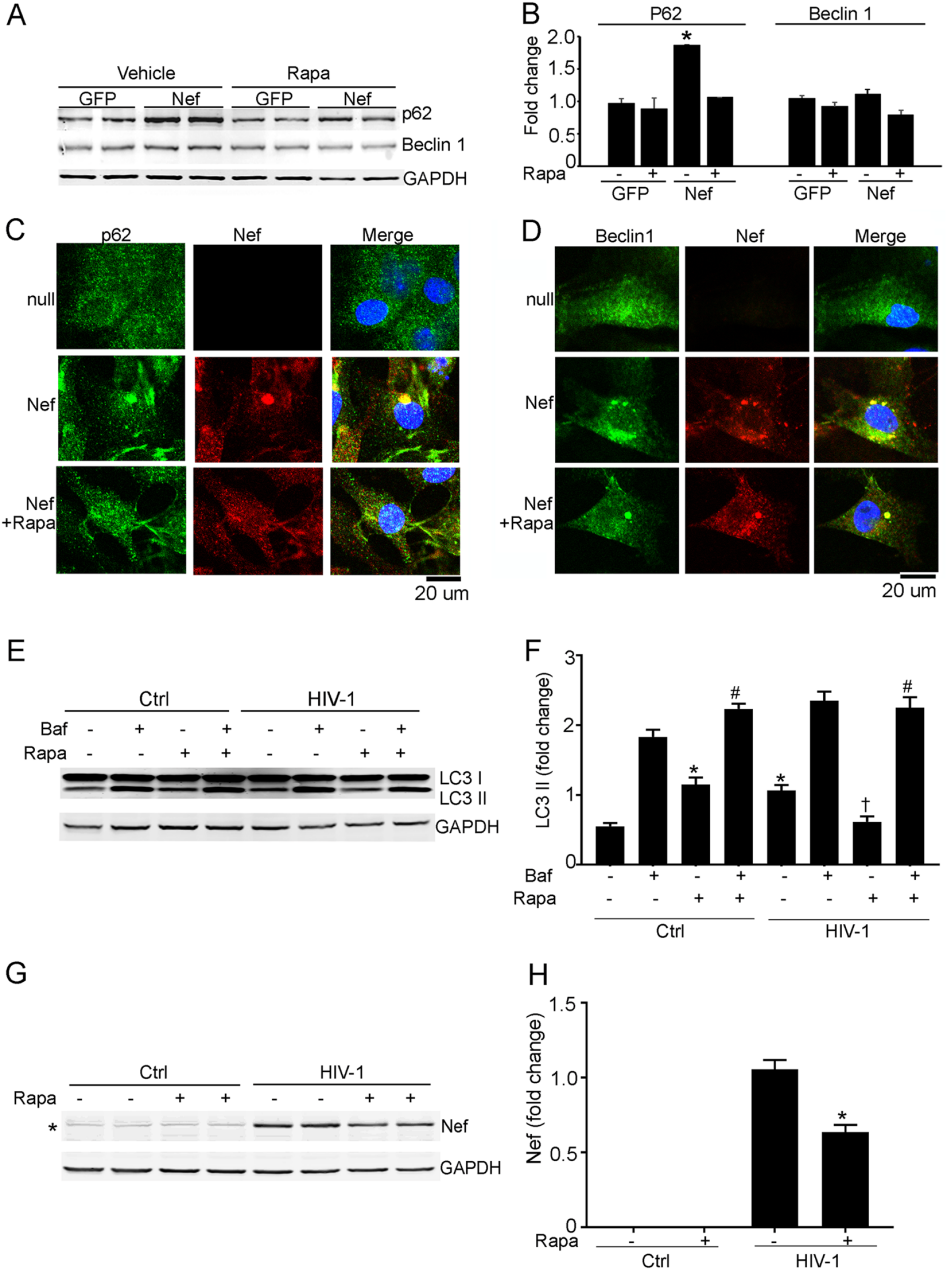
Rapamycin treatment reduced the level of aggregates in Nef-expressing cardiomyocytes. (**A**,**B**) Western blot shows that rapamycin restores the level of p62 in Nef-expressing cardiomyocytes. NRVCs were transduced with Ad-Nef or Ad-GFP for 48 hours and cells were treated with rapamycin for 12 hours before isolation of proteins (n = 6/group, *P < 0.05, significant difference between Ad-GFP and Ad-Nef). (**C**,**D**) Rapamycin treatment reduced the aggregate level of p62 and Beclin 1 in cardiomyocytes. NRVCs were transduced with Ad-Nef or Ad-null for 48 hours and cells were fixed with 4% PFA. Fixed cells were stained with p62 and Beclin 1 antibody. (**E**,**F**) Rapamycin treatment improved the autophagic flux of HIV-1-treated cardiomyocytes. Western blot showed expression of LC3-II in cardiomyocytes after rapamycin treatment. NRVCs were treated with pseudotyped HIV-1 for 5 days and also treated with rapamycin for 12 hours. Autophagy flux was determined using the autophagy inhibitor bafilomycin (n = 6/group, *P < 0.05, vs Ctrl; ^†^ *P* < 0.05, vs HIV-1, ^#^ *P* < 0.05, vs Ctrl treated bafilomycin). (**G**,**H**) Rapamycin treatment reduced the level of Nef protein in the cardiomyocytes. NRVCs were treated with HIV-1 for 5 days and also with rapamycin for 12 hours. Expression of Nef protein was determined by Western blot with Nef antibody (n = 6/group, *P < 0.05, significant difference between HIV-1 and HIV-1 treated with rapamycin). (*Indicates a nonspecific band in Western blot with Nef antibody in the HIV infected cardiomyocytes).

We tested whether rapamycin-mediated autophagy would clear Nef-induced cellular aggregates in cardiomyocytes. Rapamycin treatment restored the level of p62 protein and removed the aggregated form of p62, Beclin1 and ubiquitin from Nef-transduced cardiomyocytes (Fig. 8A–D and Supplementary Figure 9). Rapamycin treatment also reduced Nef-positive aggregates (Fig. 8C and D and Supplementary Figures 9 and 10) and restored Rab7 protein localization in Nef-expressing cardiomyocytes (Supplementary Figure 10)

We tested a potential beneficial role of rapamycin-mediated induction of autophagy in HIV-1-infected cardiomyocytes. Rapamycin significantly upregulates the autophagic flux of HIV-1-infected cardiomyocytes as seen by increase in LC3II (Fig. 8E and F). We found that rapamycin also significantly reduces the level of Nef in HIV-1-infected cardiomyocytes compared to control cells (Fig. 8G and H).

## Discussion

Prior to the introduction of combined anti-retroviral therapy (cART), a major cause of death in HIV-1 infected patients was the development of HIV-1-related cardiomyopathy. The improved viral control achieved in HIV-1 infected patients on cART has only decreased the prevalence of HIV-1-associated heart failure by about 30%, and the incidence remains unacceptably high. Little is known about the pathobiology of HIV-1 in the heart and the absence of productive infection in myocardial cells by HIV-1 has led to speculation that indirect pathways involving circulating viral and cellular factors with toxic activities are the cause of the disease. Our present findings suggest one possible mechanism for HIV-1 induced cardiomyopathy that involves the viral auxiliary protein Nef. Earlier studies suggest that Nef protein is released by HIV-1 infected cells into circulation and plasma levels can reach high concentrations of up to 10 ng/ml^30^. Similarly, we demonstrate the presence of Nef in the serum of naïve and ART-treated HIV-1 positive patients. Further, the detection of Nef in myocardial cells of the HIV-1 positive patients as well as in the hearts of SIV-infected macaques, where there is no productive viral infection, suggests that uptake of the extracellular circulating Nef by cardiomyocytes. Results from our *in vitro* cell model are consistent with this notion, as Nef was detected in the conditioned media from HIV-1 infected PBMCs and the treatment of cardiomyocytes by these media caused an array of dysregulatory events including the accumulation of an autophagy marker, LC3-II, premature formation of autophagosome and impaired autophagy flux. Autophagy plays a critical role in the maintenance of cellular protein homeostasis. Inhibition of autophagy can cause accumulation of aggregated proteins, which in turn, become cytotoxic to cells. Our results ascribed a role for Nef in dysregulating autophagy in cardiomyocytes which is manifested by the accumulation of immature autophagic vesicles and impairment of autophagy flux. Our data further demonstrate that Nef blocks autophagy at the terminal stage and consequently cells form large protein aggregates that are positive for p62 and ubiquitin.

One possible mechanism that accounts for the effect of Nef on autophagy may stem from the ability of Nef to interact with the autophagy maturation factor Beclin 1 and form aggregates in the perinuclear space. Earlier studies showed that the C-terminus of Nef strongly interacts with Beclin 1^42^. In addition, we have found that another autophagy regulatory protein, Rab7, also interacts with Nef and forms aggregates in the perinuclear space. Rab7 regulates the late stage of autophagy through promoting the fusion of autophagosome–lysosome/late endosome^58–60^. Our data show that control cardiomyocytes have isolated Rab7 punctate structures throughout the cytosol, but Rab7 forms aggregates in the perinuclear space in Nef-expressing cells and the level of soluble Rab7 is significantly decreased. Earlier, through a gain in and loss of function approach, it was demonstrated that Rab7 is critical for autophagy and inhibition of Rab7 causes impaired autophagy maturation and increased cytotoxicity. Since Rab7 plays a positive role in autophagosome maturation, we hypothesized that Nef protein inhibits the autophagy process through dysregulation of Rab7 function.

Rapamycin is known for its ability to induce autophagy through inhibition of the mTOR pathway and regulates several cellular processes including clearance of aggregated proteins and has beneficial effects in several disease models^56, 61^. Here, we observed that rapamycin-induced autophagy has beneficial effects on Nef-expressing cells and cellular aggregates significantly decreased and viability improved in rapamycin treated cells. Rapamycin treatment induces autophagy and clears aggregates of the proteins p62, ubiquitin, Beclin 1 from Nef-transduced cardiomyocytes. These results further support the notion that rapamycin treatment can neutralize Nef-mediated inhibition of autophagy. Rapamycin treatment restored the cytosolic distribution of Rab7 in cells and Nef reduced lysosome content of cells and promoted accumulation of TFEB in the cytosol. It is known that Rab7 and TFEB regulate the biogenesis of lysosomes^60, 62, 63^, therefore we hypothesize that dyslocalization of Rab7 and TFEB in Nef may cause inhibition of lysosomal biogenesis. Strikingly, we found that the treatment of Nef-expressing cardiomyocytes with rapamycin restores appropriate subcellular localization of Rab7 and TFEB, and recalibrates lysosome content in Nef-expressing cells. Furthermore, in rapamycin treated cells under stress by Nef, autophagy blockage is cleared and autophagic flux is improved.

In conclusion, our study suggests that Nef causes cardiotoxicity through inhibition of autophagy and generation of protein aggregates in cells. Nef blocks autophagy at the maturation stage of autophagosomes through inhibition of Beclin 1, Rab7 and TFEB protein accumulation. Our study further supports the notion that rapamycin might be a possible therapeutic pharmacological agent to reduce HIV-1-mediated cardiotoxicity. However, since rapamycin is widely used as an immunosuppressive agent, the clinical utility of rapamycin in treatment of HIV-1 cardiomyopathy will require further study. HIV-1 induced pathogenesis is a complex phenomenon due to changes in several cellular signaling pathways. Indeed, the pathogenic impact of Nef may further be amplified in the context of other viral, extracellular proteins including Tat, which has a toxic effect on a broad range of cells.

## Materials and Methods

### Clinical samples utilized in this study

Frozen and fixed cardiac tissues and serum: Snap frozen heart tissues and frozen serum samples from HIV patients and controls were obtained from the Manhattan HIV Brain Bank and the Texas NeuroAIDS Research Center. Formalin-fixed paraffin embedded heart tissues from HIV patients and controls were obtained from the California NeuroAIDS Tissue Network. Frozen and fixed cardiac tissues were collected at autopsy. The Manhattan HIV Brain Bank (Mt. Sinai School of Medicine, New York, NY), Texas NeuroAIDS Research Center (University of Texas Medical Branch at Galveston, Galveston, TX), and the National Neurological AIDS Bank (University of California, Los Angeles, Los Angeles, California) are all members of the National NeuroAIDS Tissue Consortium (NNTC). PBMCs were isolated from American Red Cross through Comprehensive NeuroAIDS Center Clinical Core at Temple University.

### Macaque samples utilized in this study

Archived tissues from eleven male rhesus macaques of an average age of 7.1 years and average weight of 9.7 kilograms were used in this study. Left ventricle tissues from five uninfected (SIV−) and six SIV-infected (SIV+) macaques were used. SIV+ animals were inoculated intravenously with SIVmac251 (a generous gift from Dr. Ronald Desrosiers, University of Miami) and administered 10 mg/kg of anti-CD8 antibody subcutaneously at day 6 after infection, and 5 mg/kg intravenously at days 8 and 12 after infection in order to achieve rapid AIDS. The human anti-CD8 antibody was provided by the NIH Non-human Primate Reagent Resource (RR016001, AI040101). Three of the SIV+ animals were also treated with an antiretroviral therapy consisting of tenofovir, emtricitabine (Gilead, 30 mg/kg and 10 mg/kg s.q., respectively) and raltegravir (Merck, 22 mg/kg b.i.d.) (SIV+ ART+) starting at 21 days post-infection. A full necropsy was performed when animals developed AIDS (SIV+, 56–120 days post infection) or at 120 days post infection (SIV+ ART+).

### Isolation of cardiomyocytes and cell culture

Rat primary cardiomyocytes were isolated from left ventricles of 1-2 day-old Sprague-Dawley rats (Charles River) as described previously^64^. Initially, cells were grown in MEM alpha medium with 10% FBS for 24 hours and then medium was replaced with DMEM (Gibco^TM^) with 2% FBS and gentamicin (MD).

### Isolation of PBMCs

Healthy donor PBMCs (provided by the Comprehensive NeuroAids Center (CNAC) Basic Science Core I, Temple University, Philadelphia) were isolated from buffy coat blood by density gradient centrifugation using Ficoll-Paque reagent (Sigma) as described previously^65^. Lymphocytes were cultured in RPMI (Gibco^TM^) with 10% FBS and gentamicin (10 µg/ml) (MD) supplemented with human rIL-2 at a concentration of 20 U/ml (NIH AIDS Reagent Program), and media was replaced every 2 days with fresh RPMI with 10% FBS and recombinant Interleukin 2 (rIL-2) (20 U/ml). All procedures were performed in BSL2+ lab.

### Generation of adenovirus and HIV-1 (pNL4-3-EGFP-P2A-Nef)

Adenovirus expressing Nef Sf2 (NIH AIDS reagent program) was generated as described previously^66^. Control adenovirus Ad-null and Ad-GFP were obtained from Vector Biolabs (Malvern, PA 19355). Expression of Nef protein was confirmed in NRVCs transduced with Ad-Nef by Western blotting using Nef antibody (NIH AIDS Reagent Program). Wild type HIV-1 (HIV-1 JR-FL, HIV-1 SF162, NIH AIDS Reagent Program) were prepared by infecting human PBMCs as described previously^65^. VSV-G pseudotyped HIV-1 was prepared in HEK293T cells as described previously^46, 47, 67^.

### Preparation of conditioned medium from the HIV-1 infected PBMCs

PBMCs were isolated from the blood of healthy adults as described above. Before infection cells were activated with phytohemagglutinin (PHA) for 48 hours. The activated cells were washed with RPMI and cultured at 10^6^ cell/ml with supplementary recombinant interleukin-2 (rIL2) (20 U/ml) with or without wild type HIV-1 for 3 days. Culture medium was harvested by centrifugation and cleared by filtration through a 0.2 um filter unit.

### Transduction of cardiomyocytes with adenovirus and HIV-1

NRVCs were transduced with adenovirus for 2 hours in DMEM medium with no FBS, which was replaced with DMEM plus 2% FBS as described previously^64^. For the expression of HIV-1 protein we used pseudotyped HIV-1 (pNL4-3-EGFP-P2A-Nef) as described earlier^67^. Culture supernatant was also collected for expression analysis of secreted proteins in the HIV-1 infected cardiomyocytes.

### Immunohistochemistry and microscopy

Immunocytochemistry was done as described earlier^68^. Immunohistochemistry was done to detect Nef protein in the cardiac tissue as described earlier^69^. Human clinical tissue samples were obtained from NNTC. For immunohistochemistry of frozen macaque tissues, samples were sectioned at 10 um thickness. Frozen sections were fixed with 100% cold acetone in −20 °C for 20 minutes. Frozen sections were washed in 1X PBS and blocked and stained as described earlier. Images were analyzed by confocal microscopy (Carl Zeiss 710). The following antibodies were used for cell staining: Troponin I (Millipore), Nef (NIH AIDS reagent program), LC3 (Sigma8), SQSTM1/p62 (Cell Signaling), Beclin 1 (Cell Signaling), Rab7 (Cell Signaling), TFEB (MyBioSource), Ubiquitin (Santa Cruz).

### Live cell imaging

NRVCs were grown in two well chamber slides coated with 0.1% gelatin. Cells were transduced with Ad-GFP-LC3 for 48 hours and stained with LysoTracker dye (Molecular Probes) for 30 minutes at room temperature. Images were acquired by confocal microscopy (Carl Zeiss) using a 63x oil objective. Images were analyzed using ImageJ software (NIH Image).

### Detection of Nef in clinical samples

Human patient’s serum were collected from NNTC. The concentration of HIV-1 Nef in patient serum was determined using a commercial anti-Nef ELISA kit according to manufacturer’s protocol (ImmunoDX, Woburn, MA).

### Western blot and isolation of protein

Cardiomyocytes were washed twice with cold 1X PBS and lysed with RIPA buffer (Sigma) containing 1X mammalian protease inhibitor (Sigma, P8340). Proteins in cell homogenates were separated on SDS-PAGE and transferred to nitrocellulose membrane (LI-COR). Soluble and insoluble protein fractions were prepared as reported before^68^. Antibodies against Troponin I (Millipore), Nef (NIH), LC3-II (Sigma), Rab7 (Cell Signaling), p62 (Cell signaling), Beclin 1 (Cell Signaling, 3495) Akt (Cell Signaling, 9272) and pAkt (Cell Signaling), GFP (Clontech), Actinin (Sigma) were used for the Western blot analysis.

### Immunoprecipitation Assay

HEK293T cells were transfected with pQBI-nefGFP (Quantum Biotechnologies, AFP3203 and pEGFP-C1 (Clontech) plasmid and cells were grown for another 48 hours. Total proteins were isolated using RIPA with protease inhibitor. For interaction studies, proteins were precipitated with GFP antibody and immunoprecipitates were probed with Beclin 1, Rab7, Nef and GFP antibodies as described previously^64^.

### Protein extraction from cell culture media

Total culture media protein was isolated by TCA precipitation as described previously^70^. In brief, culture media protein was isolated from dead cells and cell debris by centrifugation at 3000 g. Then supernatant was collected and passed through 0.2 μm filter. Proteins were precipitated by addition of TCA (Sigma) in 1:4 ratio and incubation on ice. Proteins were isolated by centrifugation at 14,000 rpm. Precipitated protein was washed with ice cold acetone and dried at room temperature. Dry proteins were dissolved in RIPA buffer.

### Determination of autophagy

We determined cellular autophagy using autophagy marker protein LC3-II and SQSTM1/p62 protein expression. NRVC cells were transduced with Ad-Nef for 48 hours and expression of autophagy marker proteins determined through Western blot. For the determination of autophagy flux, cells were treated with bafilomycin (Sigma) at concentration of 50 nM, and expression of LC3-II determined by Western blot. We also determined the autophagy flux of cardiomyocytes using autophagy reporter system Ad-tfLC3^52^. The yellow and red marker protein expression indicates the status of the cellular autophagosome and autophagosome-lysosome fusion, respectively. For the induction of autophagy, cells were treated with rapamycin (Sigma) at a concentration of 50 nM for 12 hours before analysis.

### Determination of cellular viability and cell death

Cellular viability and cytotoxicity was determined by plating NRVCs in 96 well plates at a density of 10,000 cells per well. Nef proteins were expressed using Ad-Nef, with Ad-null as a control, for 48 hours. Cellular cytotoxicity was determined using SYTOX Green (Life technologies) and cell death assay, and cellular viability was determined using CellTiter blue (Promega). Cell death in cell cultures was determined by PI and Hoechst 33342 (Life Technologies) staining as described previously^64^.

### Data analysis and statistical procedures

Data are presented as mean ± SD. Statistical analyses between the experimental groups were performed by one-way or two-way analysis of variance (ANOVA) and the data was tested through Tukey’s test or Dunnett’s test. The threshold of statistical significance for all tests was defined at p < 0.05.

### Human subjects ethics statement

All experiments were performed in accordance with relevant guidelines and regulations. All relevant studies have been reviewed and approved by the Katz School of Medicine at Temple University Institutional Review Board. All patient samples were obtained under informed consent and approved by their local Institutional Review Board (IRB). Written informed consent for frozen and fixed cardiac tissues and serum was obtained and is maintained by the Manhattan HIV Brain Bank (Mt. Sinai School of Medicine, New York, NY), Texas NeuroAIDS Research Center (University of Texas Medical Branch at Galveston, Galveston, TX), and the National Neurological AIDS Bank (University of California, Los Angeles, Los Angeles, California), members of the National NeuroAIDS Tissue Consortium (NNTC) under local IRB- approved ethical guidelines from all subjects or their primary next-of-kin. De-identified samples were provided by the NNTC member sites from HIV patients with no history of ART treatment, HIV patients on ART treatment, and HIV negative controls.

### Vertebrate animals ethics statement

All macaques were handled in strict accordance with American Association for Accreditation of Laboratory Animal Care with the approval of the Institutional Animal Care and Use Committee of Tulane University (protocol number P0263) and housed at the Tulane National Primate Research Center (TNPRC, Covington, LA). Primary cultures of rat neonatal cardiomyocytes were obtained from the Comprehensive NeuroAIDS Center Basic Science Core I under protocols approved by the Temple University Institutional Animal Care and Use Committee (IACUC).

## Electronic supplementary material

Supplementary Information
