# Supplementary material for: HIV-1 Nef-induced cardiotoxicity through dysregulation of autophagy
Source: Sci Rep. 2017 Aug 17;7:8572. doi: 10.1038/s41598-017-08736-x (PMC5561171; doi:10.1038/s41598-017-08736-x)

3/10/17

HIV-1 Nef-induced cardiotoxicity through dysregulation of autophagy

SUPPLEMENTARY MATERIAL

Manish K Gupta[1] PhD, Rafal Kaminski[1] PhD, Brian Mullen[1] BS, Jennifer Gordon[1] PhD, Tricia H Burdo[1] PhD, Joseph Y Cheung[2,3] MD, PhD, Arthur M Feldman[2,4] MD, PhD, Muniswamy Madesh[3,5] PhD, Kamel Khalili[1] PhD[*]

[1]Department of Neuroscience, Center for Neurovirology and Comprehensive NeuroAIDS Center, [2] Department of Medicine, [3]Center for Translational Medicine, [4]Cardiovascular Research Center, [5]Department of Medical Genetics and Molecular Biochemistry, Lewis Katz School of Medicine at Temple University, Philadelphia PA, USA

SUPPLEMENTARY FIGURE 1

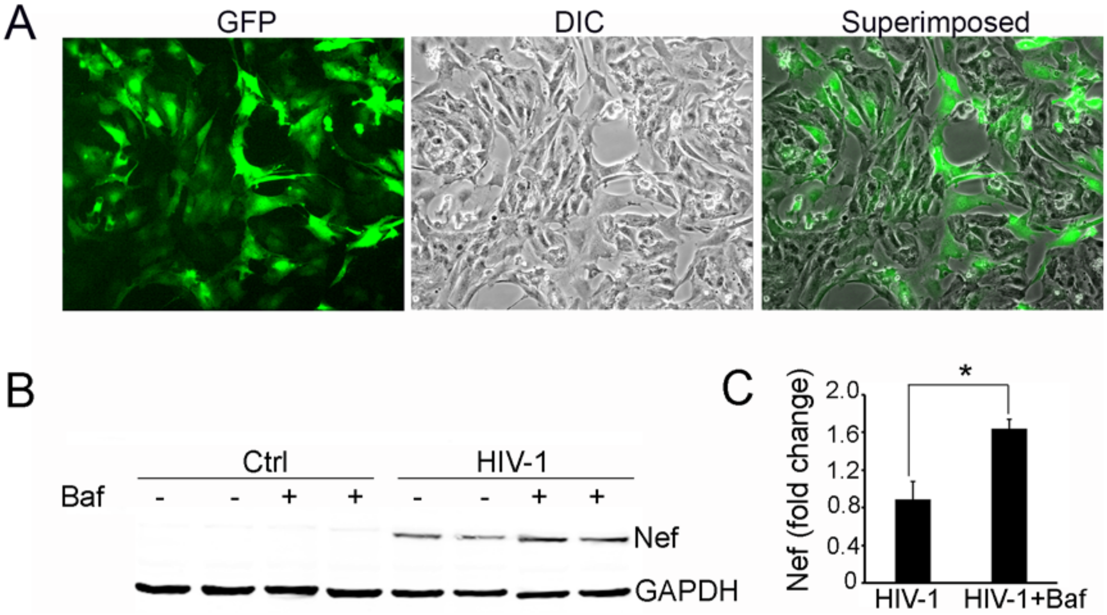

Supplementary Figure 1. Inhibition of autophagy causes accumulation of Nef protein in the cell. (A) NRVCs were infected with HIV-1 for 5 days and infected cells are visualized as a GFP positive cells (B-C) Western blot show that inhibition of autophagy by bafilomycin resulted in Nef protein accumulation in HIV-1-infected cardiomyocytes. (n=6/group, *$P<0.05$).

SUPPLEMENTARY FIGURE 2

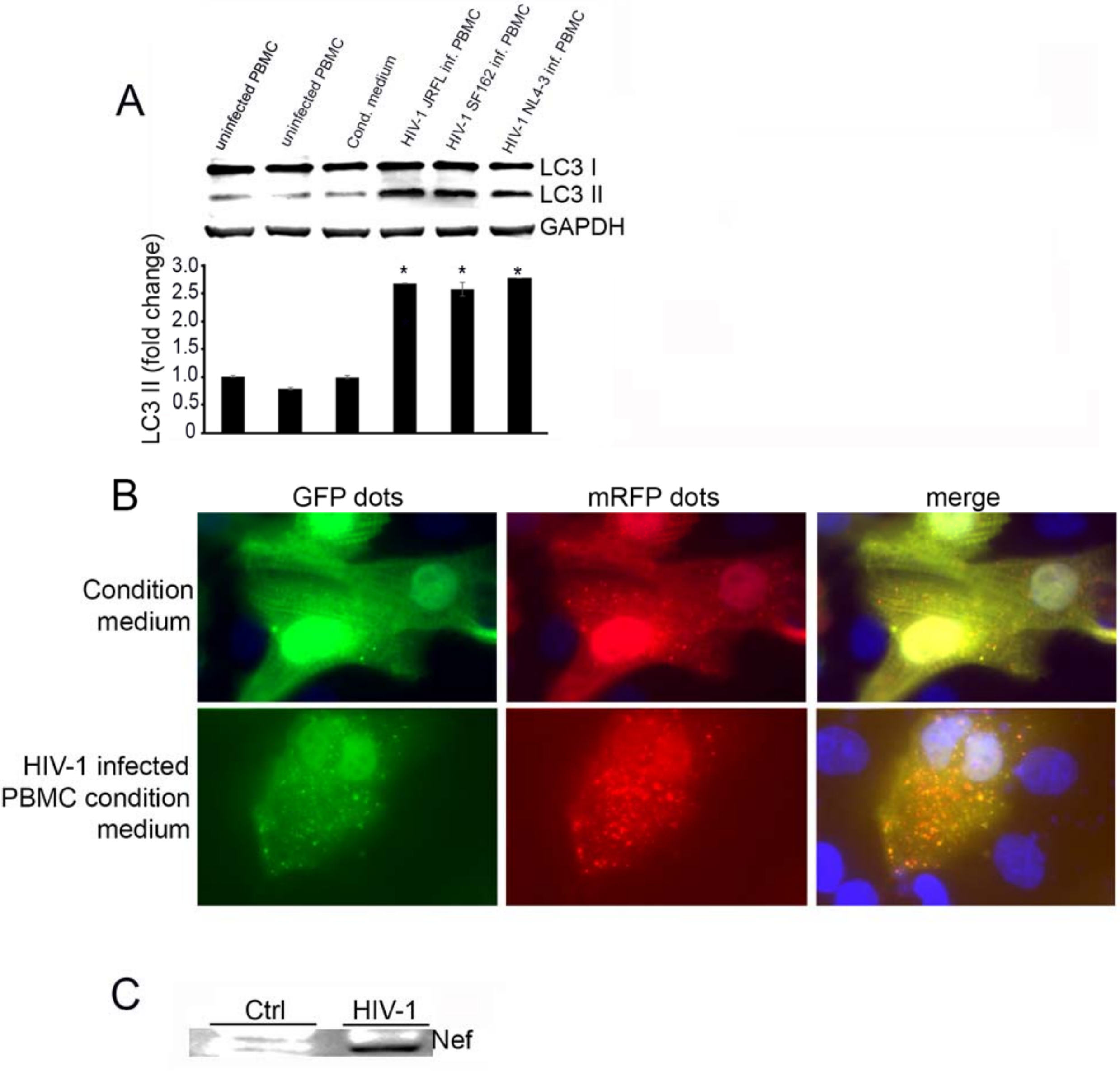

Supplementary Figure 2. Conditioned medium from wild-type HIV-1-infected PBMC decreases the autophagy in cardiomyocytes. (A) Western blot shows accumulation of LC3-II in cardiomyocytes treated with HIV-1-infected PBMCs. NRVCs were treated with conditioned medium isolated from HIV-1-infected PBMCs for 12 hours. Control cells were treated with control condition medium from uninfected PBMCs. Autophagy marker protein expression LC3-II was detected by Western blot with LC3 antibody (n=3/group, **P*<0.05 vs condition medium). (B) Representative images showing the autophagy puncta (green and red dot) in the

cardiomyocytes treated with conditioned medium. Conditioned medium from HIV-1-infected PBMCs causes accumulation of premature autophagosomes (yellow puncta) in the cardiomyocytes. NRVC cells were transduced with autophagy reporter virus Ad-tfLC3 and treated with conditioned medium for 12 hours. Images were captured from fixed cells under fluorescence microscope. (C) Western blot shows the presence of Nef protein in the culture medium of HIV-1 infected PBMCs.

SUPPLEMENTARY FIGURE 3

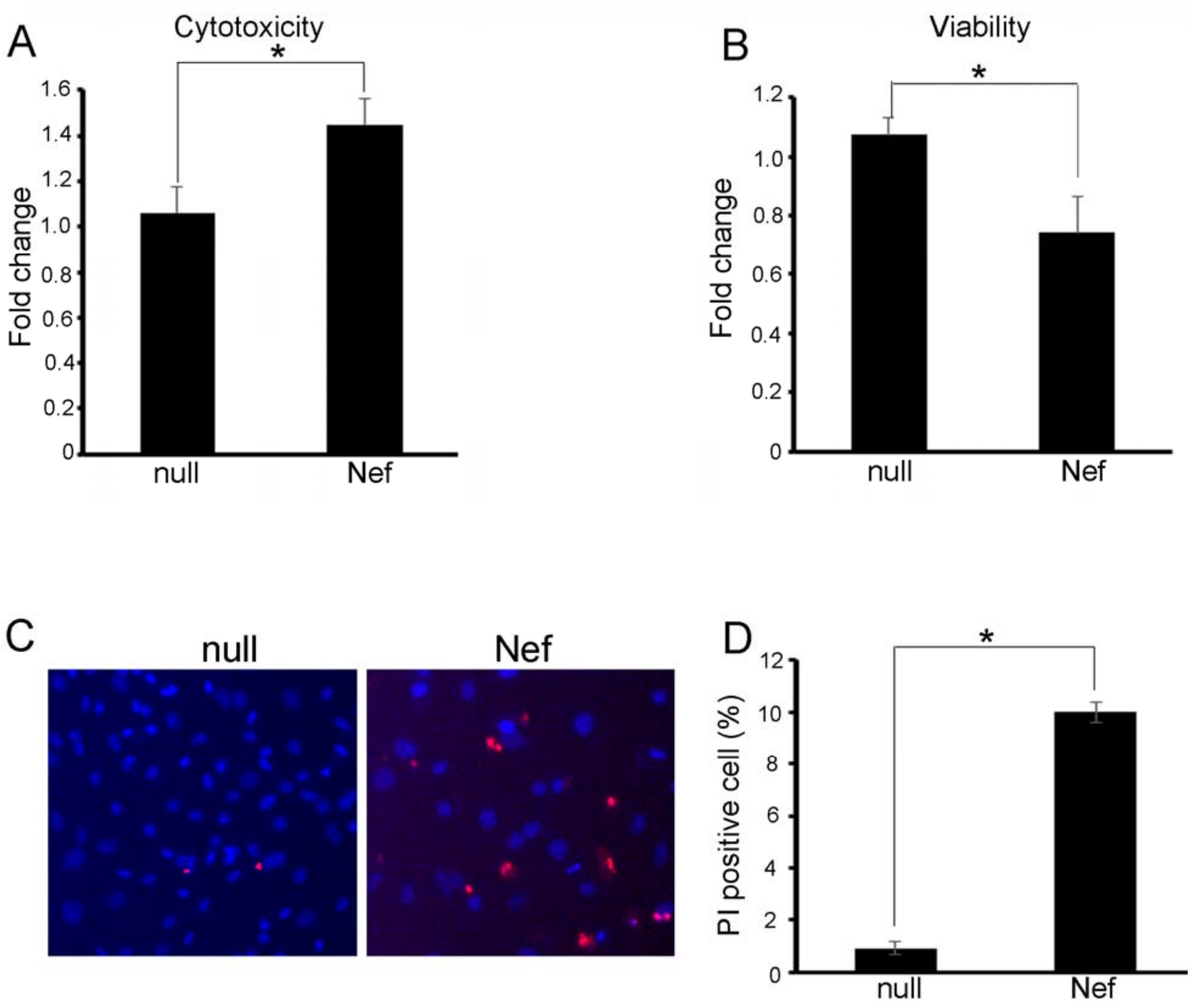

Supplementary Figure 3. Nef causes cell death through induction of cytotoxicity and viability loss. (A-B) NRVCs were transduced with Ad-Nef or Ad-null for 48 hours and cellular cytotoxicity and viability was determined using CYTOX green and CellTiter-Blue respectively (n=20/group, *P<0.05 significance different Ad-null vs Ad-Nef). (C-D) NRVCs were transduced with Ad-Nef for 48 hours and cell death was determined by live imaging after staining cells with PI (red) and Hoechst 33342 (blue) (n=4 well/group, *P<0.05 significant difference between Ad-null vs Ad-Nef).

SUPPLEMENTARY FIGURE 4

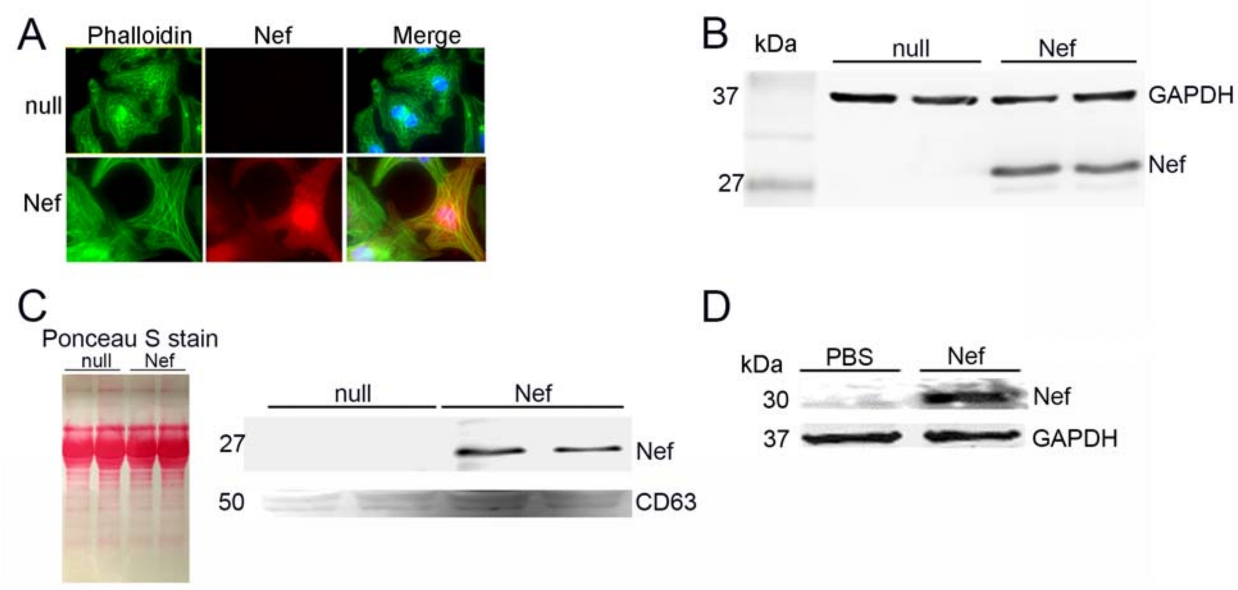

Supplementary Figure 4. Nef protein localizes to the perinuclear space. (A) Representative images shows that Nef protein localizes to the perinuclear space in the rat cardiomyocytes. NRVCs were transduced with Ad-Nef for 48 hours and cells were fixed with 4% PFA. Fixed cells were stained with phalloidin and Nef. (B) Western blot show that expression of Nef protein in cardiomyocytes after 48 hours of Ad-Nef transduction. (C) Western blot shows presence of Nef protein in the culture medium of NRVCs transduced with Ad-Nef for 48 hours.. Total culture filtrate proteins were visualized in the nitrocellulose membrane by Ponceau S staining and probed with Nef and CD63 antibody respectively. (D) Western blot show that recombinant Nef protein is present in cardiomyocytes. NRVCs were incubated with 4 hours in the DMEM having recombinant Nef protein.

SUPPLEMENTARY FIGURE 5

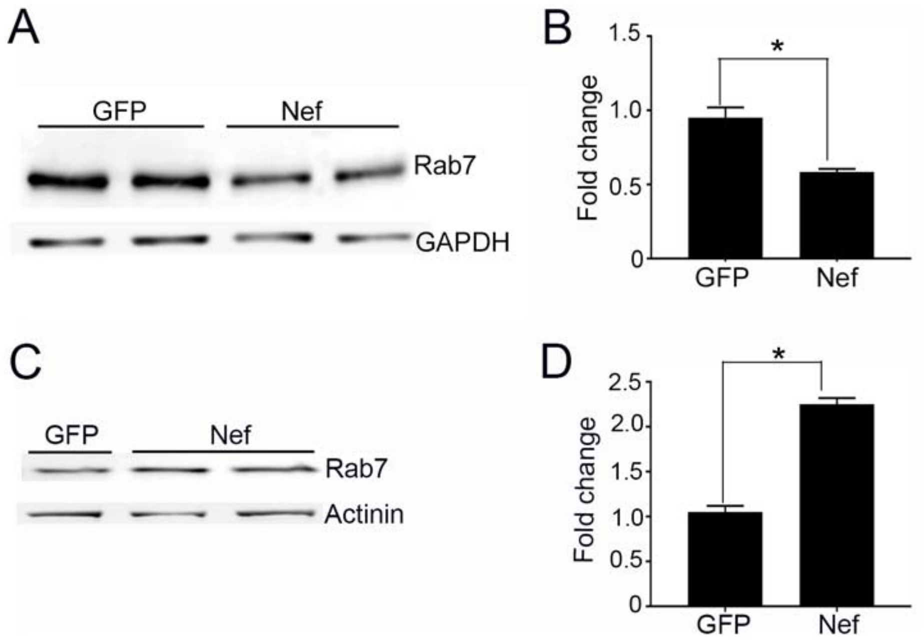

Supplementary Figure 5. Expression of Nef protein alters distribution of Rab7 between aggregate and non-aggregate form. Western blots show expression of Rab7 in the NRVCs. Cardiomyocytes were transduce with Ad-Nef for 48 hours and Rab7 protein level was detected by Western blot in soluble (non-aggregate) (A-B) and insoluble fraction (aggregate) (C-D). Blots were probed with Nef antibody and GAPDH as loading control in soluble fraction and actinin in insoluble fraction (n=6/group, **P*<0.05).

SUPPLEMENTARY FIGURE 6

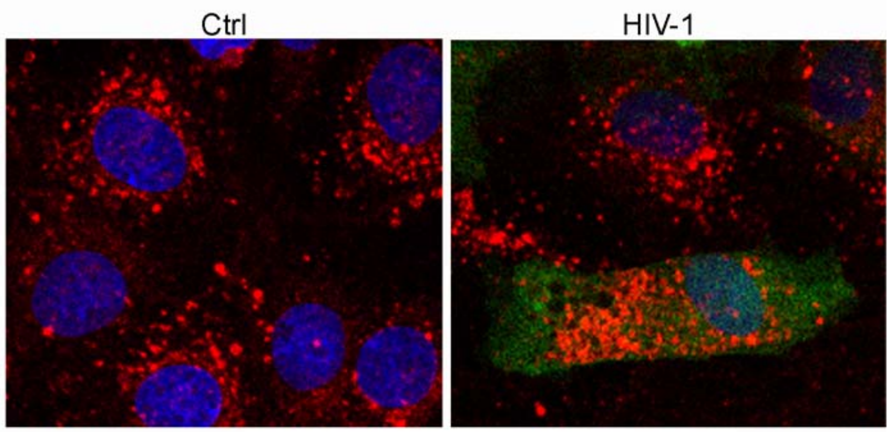

Supplementary Figure 6. HIV-1 infected cardiomyocytes caused dysregulation of Rab7 protein localization. Representative images show that Rab7 protein forms aggregate in the HIV-1 infected cardiomyocytes. NRVCs were infected with pseudotyped HIV-1 for 3 days. Cells were fixed with 4% PFA and stained with Rab7 antibody (red) and HIV-1 infected cells are positive for GFP (green).

SUPPLEMENTARY FIGURE 7

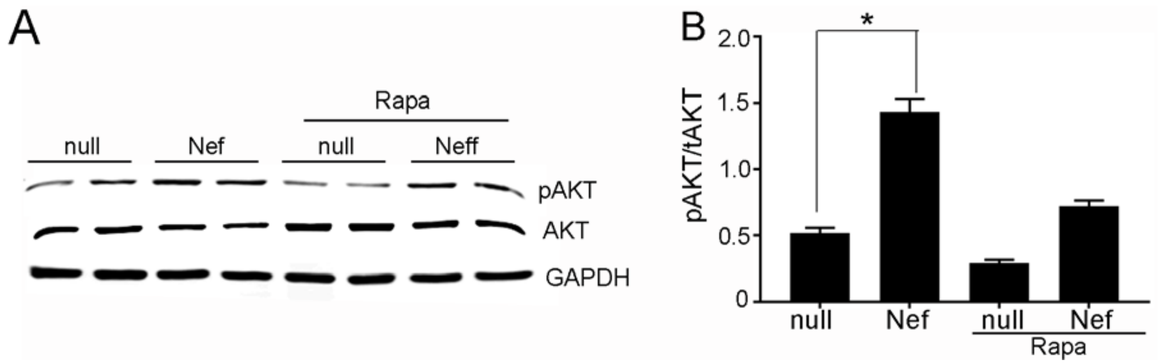

Supplementary Figure 7. Rapamycin treatment reduces the level of phosphorylated Akt in Nef expressing cardiomyocytes. (A-B) Western blot shows the expression of Akt and phospho-Akt in cardiomyocytes transduced with Ad-Nef. NRVCs were transduced with Ad-Nef or Ad-null for 48 hours and Western blot was done with total cell protein lysate using Akt and phospho-Akt antibody respectively (n=4 group, **P*<0.05).

SUPPLEMENTARY FIGURE 8

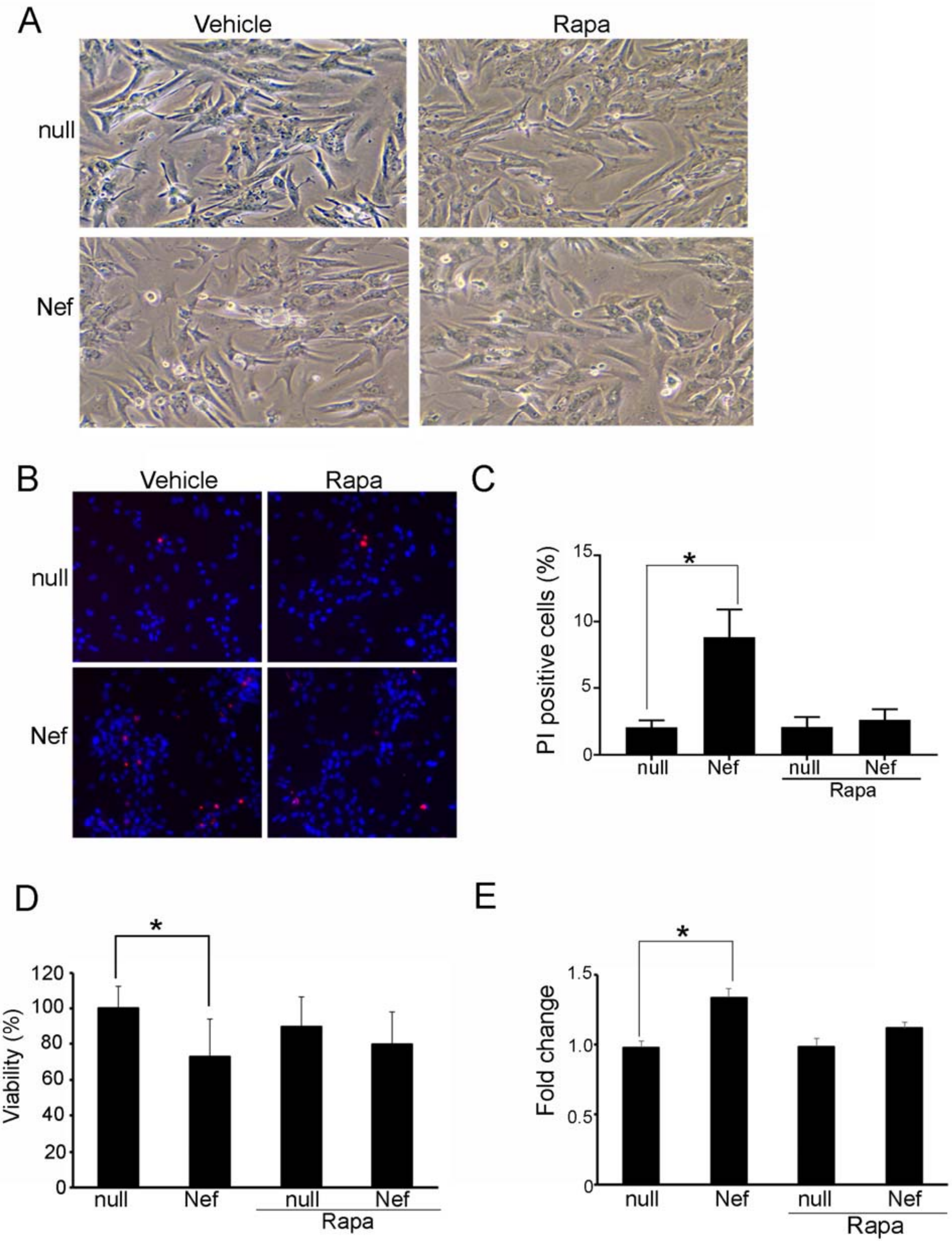

Supplementtary Figure 8. Rapamycin treatment reduces the cell death and improves cellular viability of Nef-expressing cardiomyocytes. (A) Representative images show that Nef expression causes change in the cellular morphology and apoptotic body formation in cardiomyocytes. (B-C) Representative images show that Nef expressing cells have higher number of cell death and rapamycin treatment reduces the cells death. NRVC cells were stained with PI (red) and Hoechst (blue) (n=4 well/group, *$P<0.05$). (D-E) Rapamycin treatment improves cellular viability of Nef-expressing cells and reduces cellular toxicity. Nef protein was expressed in cardiomyocytes for 48 hours and cellular viability and cytotoxicity were determined using CellTiter blue and SYTOX green respectively (n=20/group, *$P<0.05$).

SUPPLEMENTARY FIGURE 9

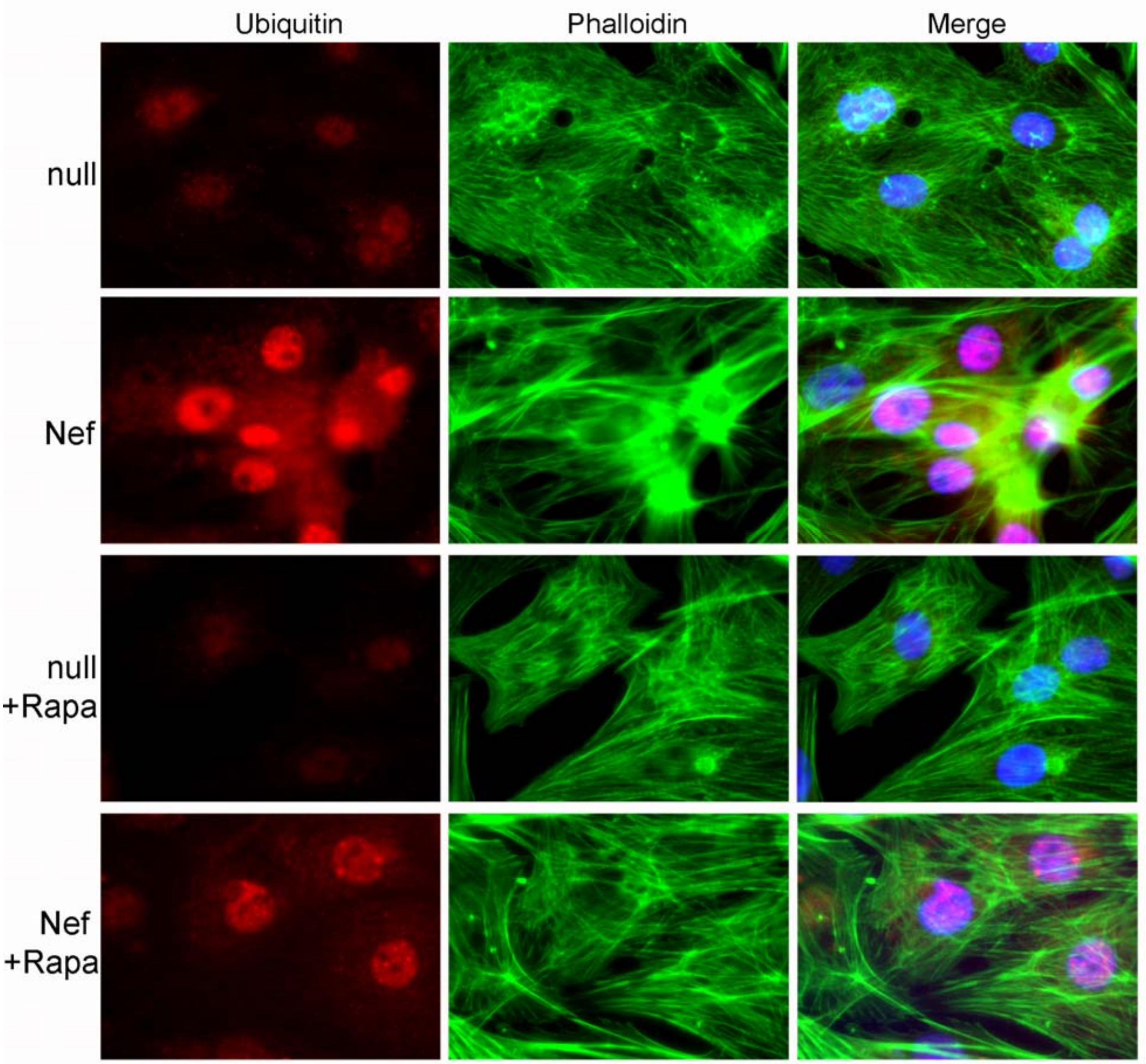

Supplementary Figure 9. Rapamycin treatment reduced the level of ubiquitin-positive aggregates in Nef-expressing cardiomyocytes. Representative images show the level of ubiquitin-positive protein in the cardiomyocytes after Nef expression. NRVCs were transduced with Ad-Nef for 48 hours and rapamycin treatment done for 12 hours before cell fixation. Cells were fixed with 4% PFA and fixed cells were stained with phalloidin (green) and ubiquitin antibody (red).

SUPPLEMENTARY FIGURE 10

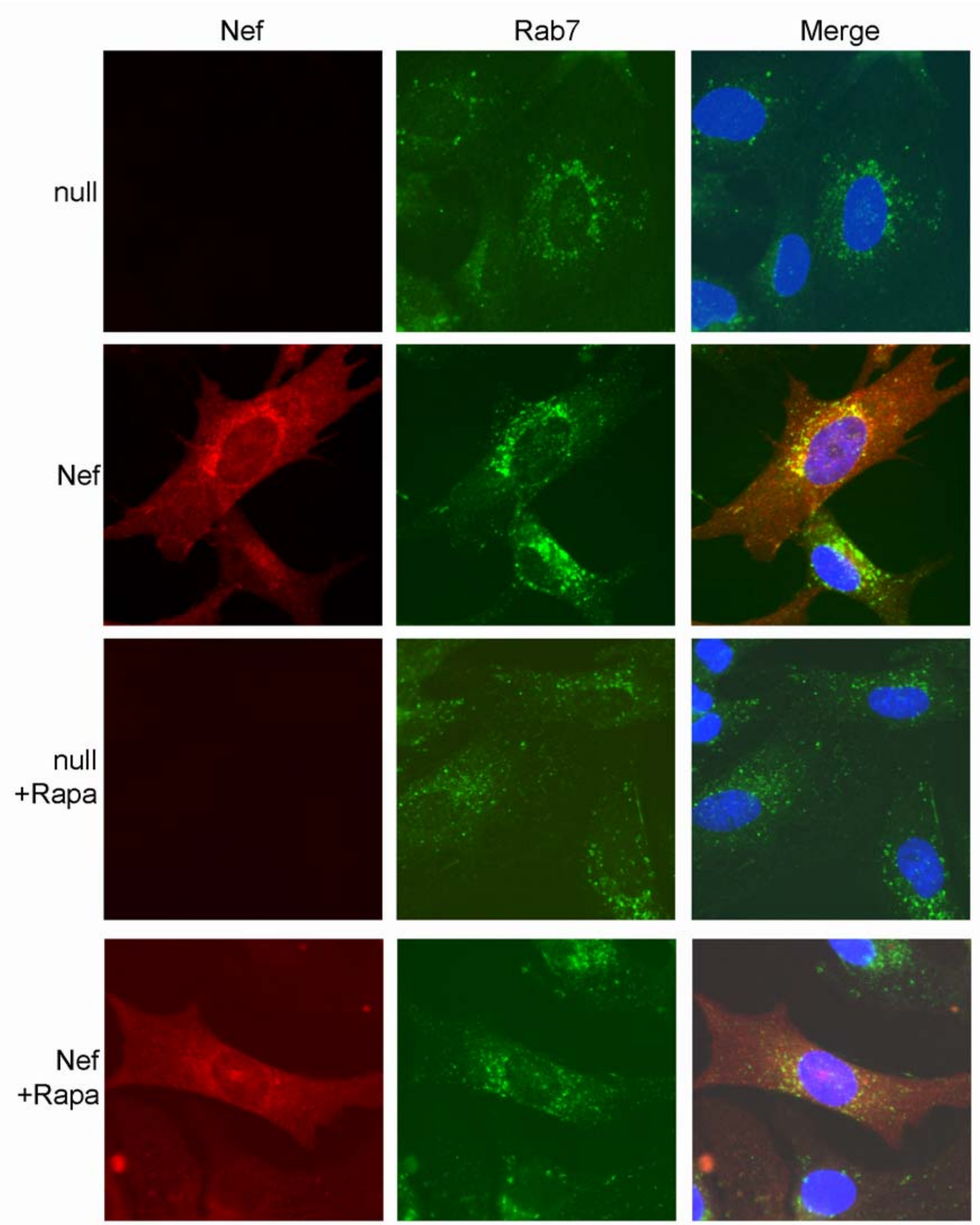

Supplementary Figure 10. Rapamycin treatment reduces the Rab7 aggregate in Nef expressing cardiomyocytes. Representative images showing the distribution of Rab7 and Nef protein in the cardiomyocytes. NRVCs were transduced with Ad-Nef or Ad-null for the 48 hours

and treated with rapamycin for 12 hours before cell fixation. Cells were fixed with 4% PFA and stained with Nef (red) and Rab7 (green) antibody.

HIV-1 Nef-induced cardiotoxicity through dysregulation of autophagy

Manish K. Gupta, PhD, Rafal Kaminski PhD, Brian Mullen, BS,

Jennifer Gordon, PhD, Tricia H. Burdo, PhD, Joseph Y. Cheung, MD, PhD,

Arthur M. Feldman, MD, PhD, Muniswamy Madesh, PhD, Kamel Khalili, PhD

Uncropped Figures

Figure 1B

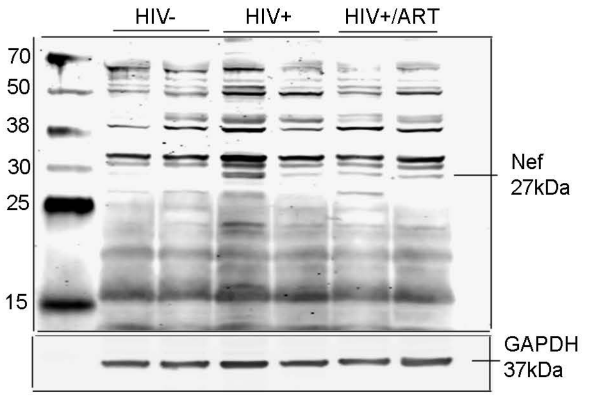

Figure 1D

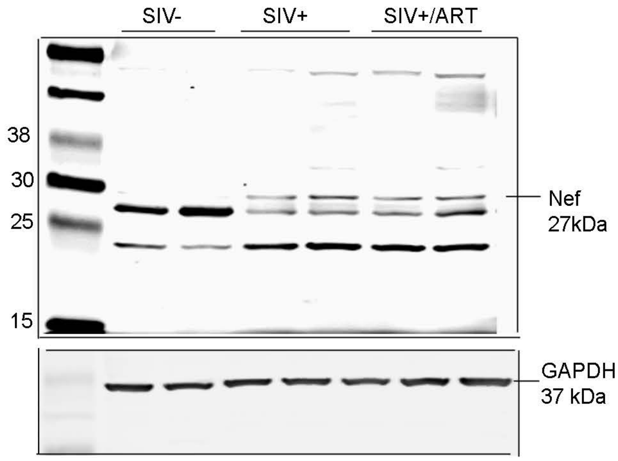

Figure 2G

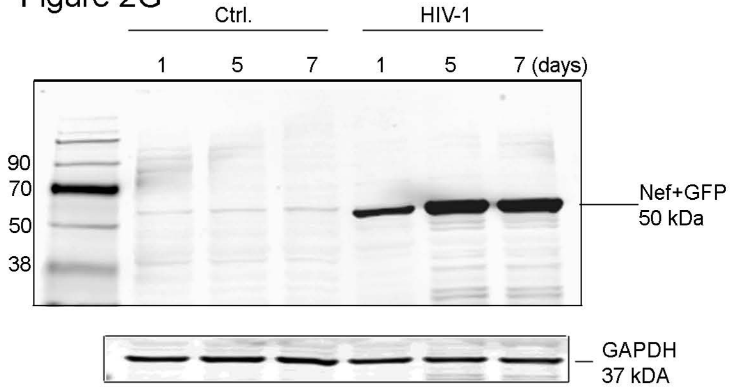

Figure 2H

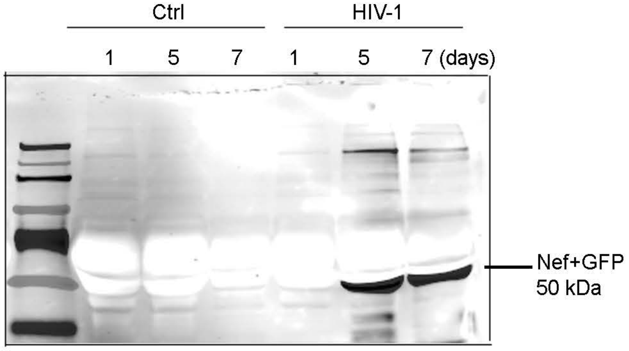

Figure 2I

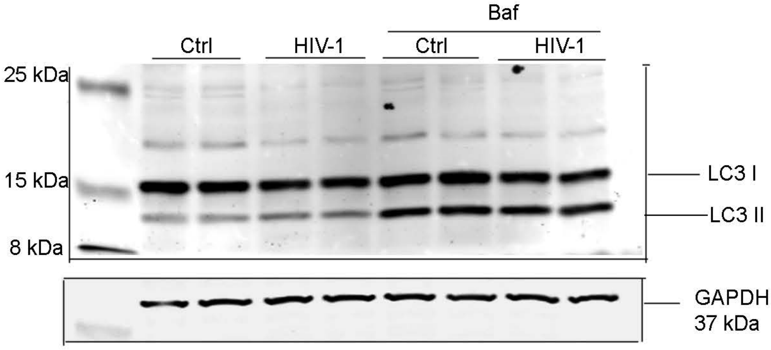

Figure 2K

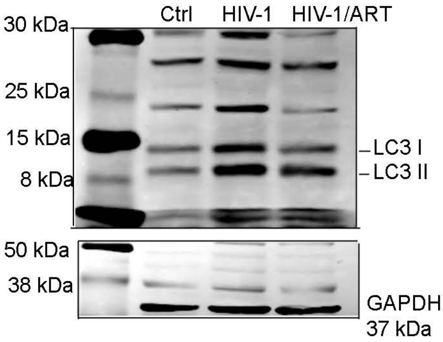

Figure 3A

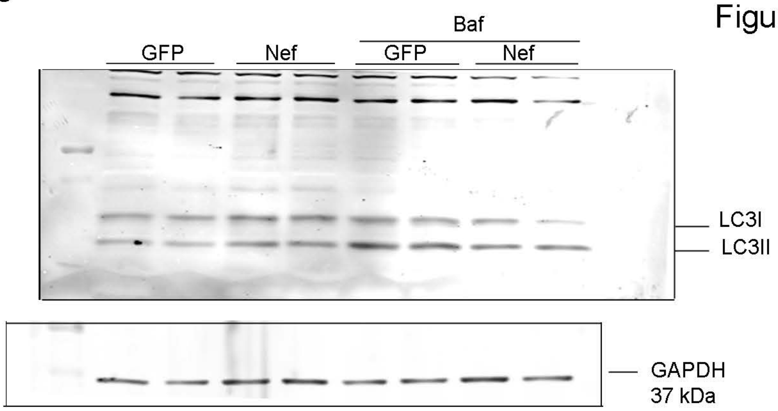

Figure 4D

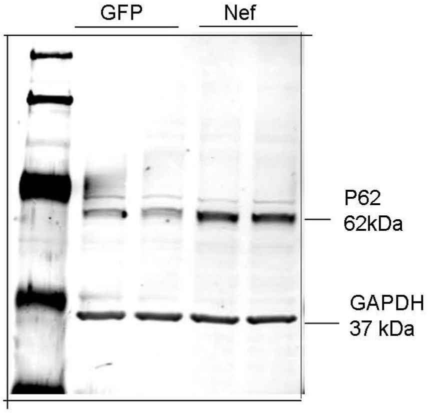

Figure 6A

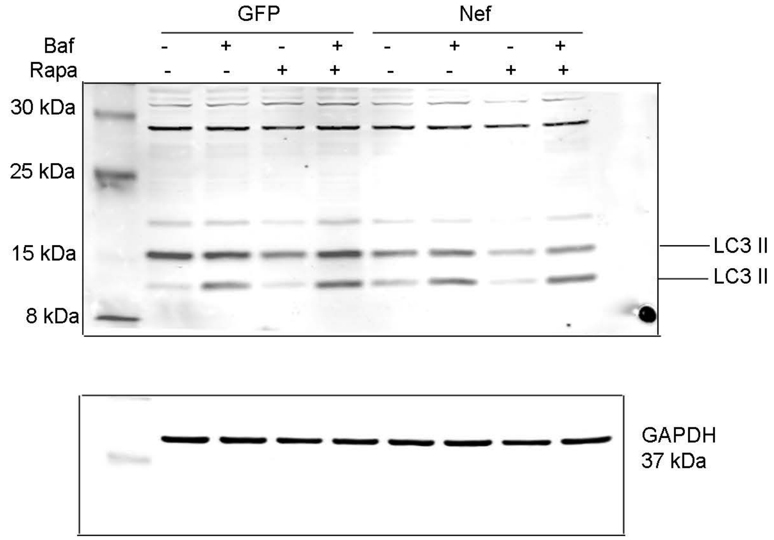

Figure 8A

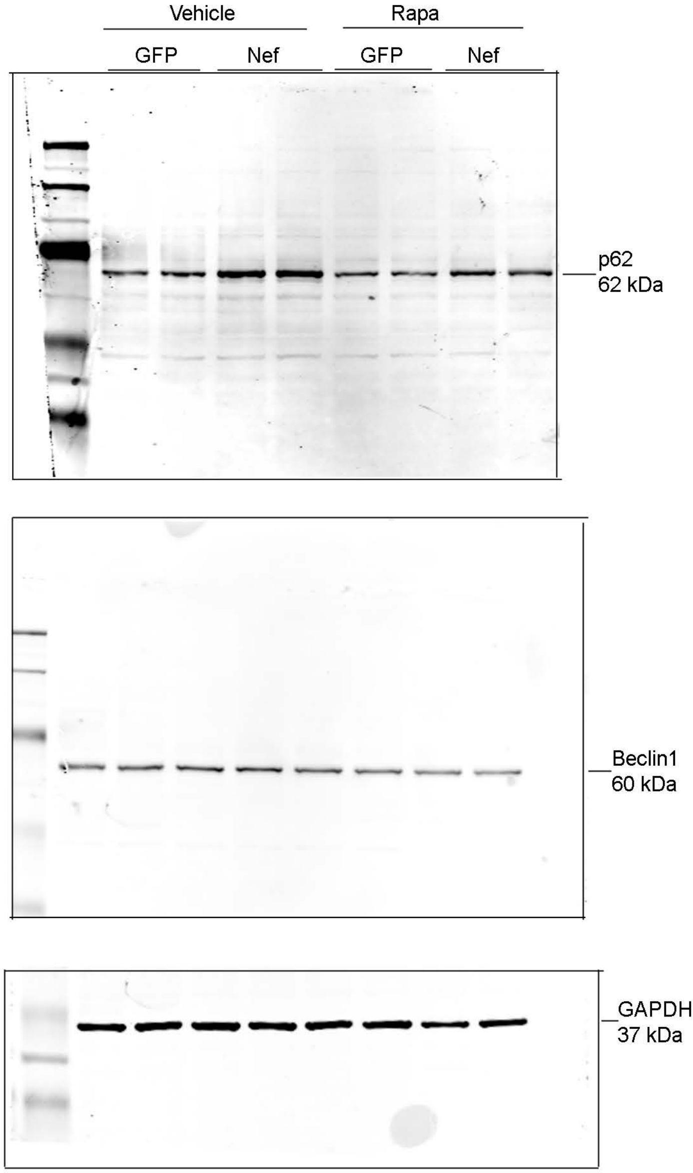

Figure 8E

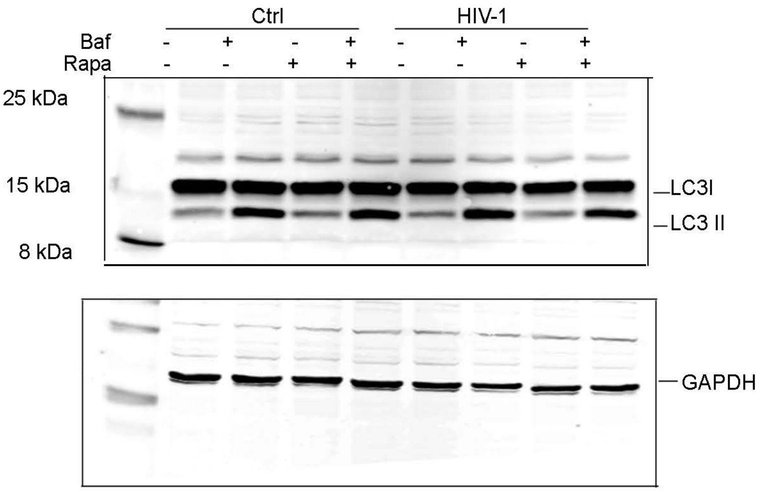

Figure 8G

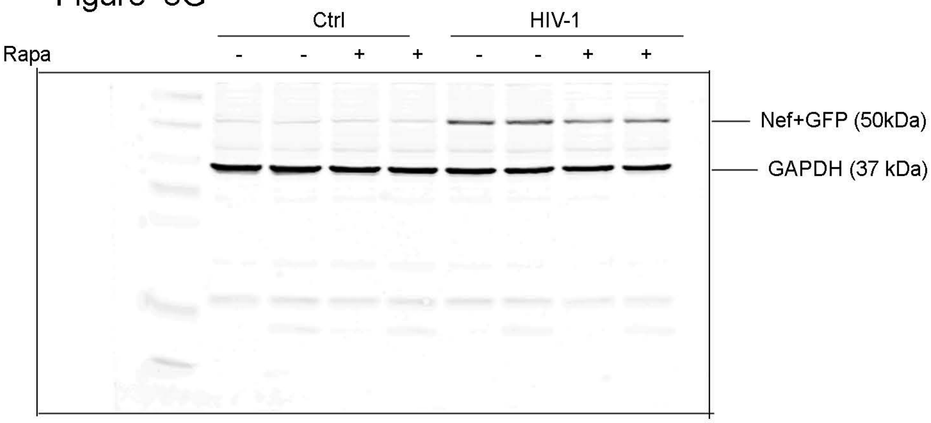

Supplementary Figure 1B

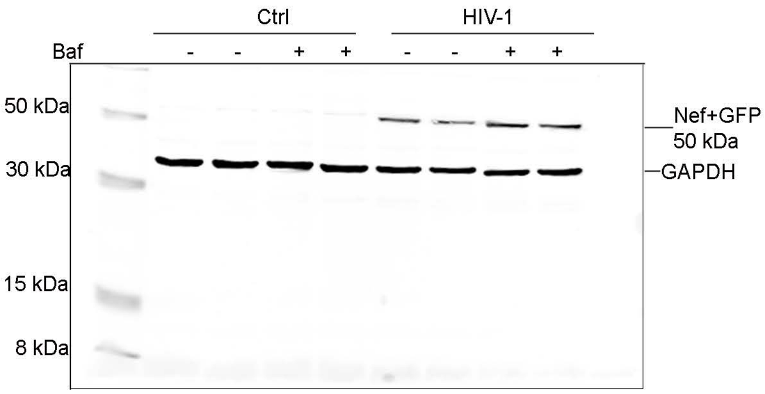

Supplementary Figure 2A

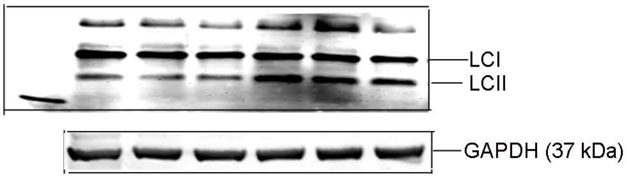

Supplementary Figure 4C

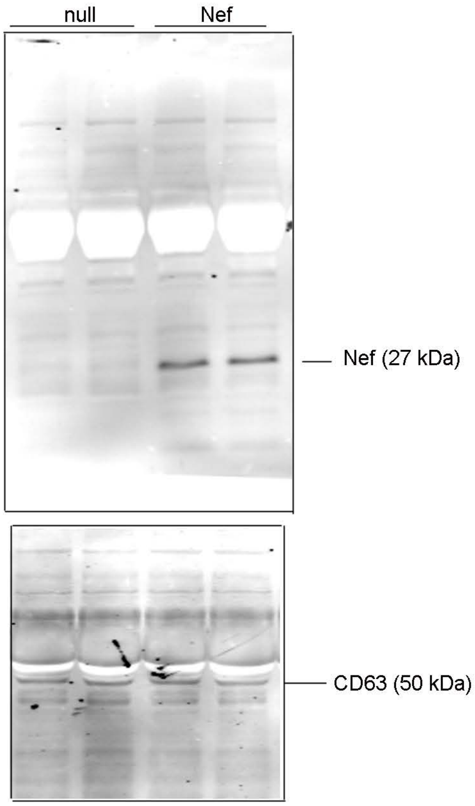

Supplementary Figure 2C

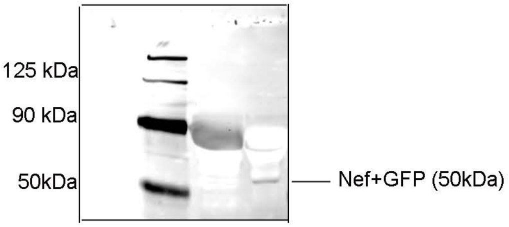

Supplementary Figure 5A

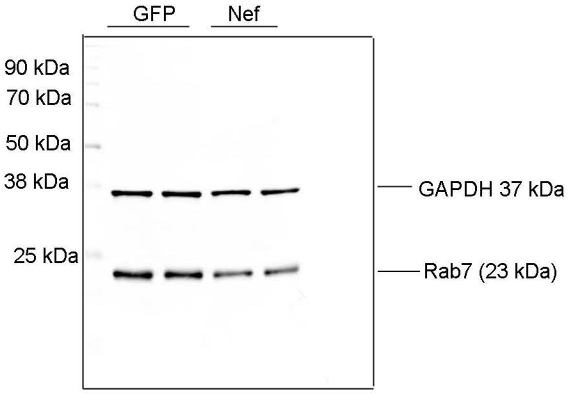

Supplementary Figure 7A

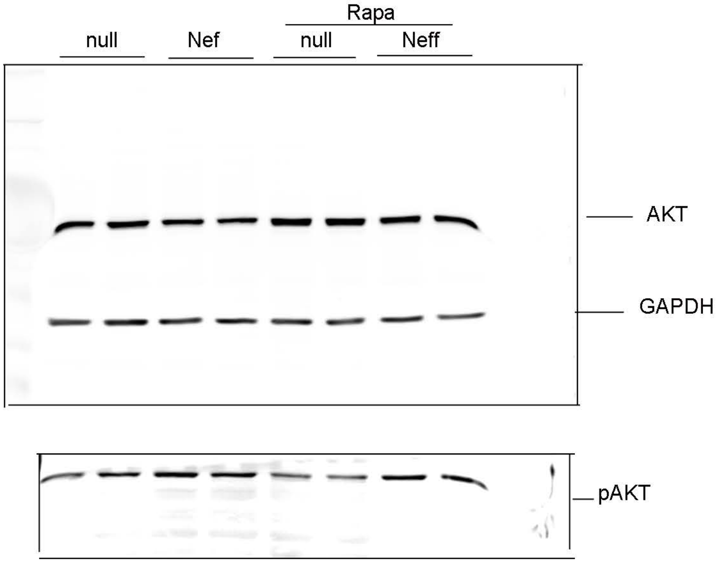

Supplementary Figure 5C

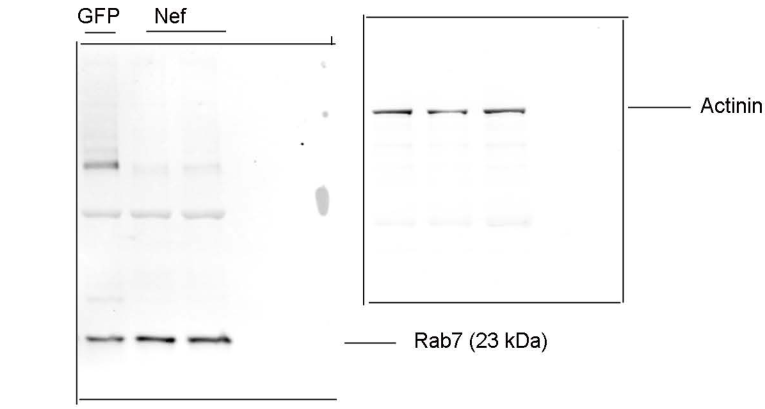

Supplement: Supplementary file 1 — Supplementary Information [file 41598_2017_8736_MOESM1_ESM.pdf]
